# Cisplatin Resistance in Epstein–Barr-Virus-Associated Gastric Carcinoma Acquired through *ATM* Methylation

**DOI:** 10.3390/cancers13174252

**Published:** 2021-08-24

**Authors:** Sun Hee Lee, Su Jin Choi, Wonhyeok Choi, Subin Cho, Miyeon Cho, Dong Sun Kim, Byung Woog Kang, Jong Gwang Kim, You Mie Lee, Hyosun Cho, Hyojeung Kang

**Affiliations:** 1 Vessel-Organ Interaction Research Center, VOICE (MRC), Cancer Research Institute, College of Pharmacy, Kyungpook National University, Daegu 41566, Korea; ihappy278@nate.com (S.H.L.); sujinchoi88@naver.com (S.J.C.); tnqls5019@naver.com (S.C.); cmy1004g@naver.com (M.C.); 2 Duksung Innovative Drug Center, College of Pharmacy, Duksung Women’s University, Seoul 01369, Korea; dnjsgur8367@naver.com; 3 Department of Anatomy, School of Medicine, Kyungpook National University, Daegu 41944, Korea; doskim@knu.ac.kr; 4 Department of Oncology/Hematology, Cancer Research Institute, School of Medicine, Kyungpook National University, Kyungpook National University Hospital, Daegu 41405, Korea; bwkang@knu.ac.kr (B.W.K.); jkk21c@knu.ac.kr (J.G.K.); 5 Vessel-Organ Interaction Research Center, VOICE (MRC), Department of Molecular Pathophysiology, College of Pharmacy, Kyungpook National University, Daegu 41566, Korea; lym@knu.ac.kr

**Keywords:** cisplatin, 5-Azacytidine, DNA methylation, Epstein–Barr virus, DNMT3A, ATM

## Abstract

**Simple Summary:**

Gastric cancer (GC) is the fifth-leading type of cancer and the third –leading cause of death from cancer. Epstein-Barr virus-associated gastric carcinoma (EBVaGC) is recently accountable for 10% of all the GC worldwide. Platinum drugs such as cisplatin and oxaliplatin are the first-line choice in GC chemotherapy. The widespread use of cisplatin leads to make tumor cells develop single or multiple drug resistance via various mechanisms. DNA hypermethylation on tumor suppressor genes is one of causes leading to drug resistances. 5-Azacytidine (5-AZA) is a chemical analogue of cytidine and inhibits DNA methyltransferase, resulting in DNA hypomethylation. Our main objective was to identify synergistic effect of two important GC drugs whose mechanisms may be in complementary cooperation. We found that cisplatin enhances its anticancer activity with 5-AZA through DNA demethylation in EBVaGC. Identifying this synergistic effect of two important GC drugs can be useful to treat EBVaGC which shows resistance to platinum-based chemotherapy.

**Abstract:**

Epstein–Barr-virus-associated gastric carcinoma (EBVaGC), first reported in 1992, currently accounts for 10% of all gastric carcinoma worldwide. EBVaGC has unique DNA hypermethylation phenotypes that allow for higher proportions of DNA methylation than any other gastric cancer. CpG islands in the gene promoter region are one of the major regions in which DNA methylation controls gene transcription. Despite cisplatin-based chemotherapy being one of the standard treatment regimens for advanced gastric cancer, including EBVaGC, cisplatin alone or in combination with 5-fluorouracil has been limited by its less potent anticancer activity and the occurrence of cisplatin resistance. Accordingly, the current study evaluated the anticancer activities of a combination of cisplatin and 5-Azacytidine (5-AZA) against EBVaGC. Our findings showed that cisplatin upregulated the *DNMT3A* gene, whereas shRNA-targeted removal of *DNMT3A* mRNA contributed to cisplatin-mediated EBV lytic reactivation. Moreover, the removal of *DNMT3A* mRNA upregulated the *ATM* gene through DNA demethylation on the *ATM* promoter. Furthermore, CRISPR/Cas9-targeted removal of the *ATM* gene resulted in significantly reduced cell susceptibility and EBV lytic reactivation by a combination of cisplatin and DNMT3A inhibitor 5-AZA. Finally, 5-AZA exhibited a synergistic effect with cisplatin in anti-EBV and anti-EBVaGC activities by increasing drug susceptibility and EBV lytic reactivation. The aforementioned results suggest that cisplatin combined with DNA methylation inhibitors could be a novel therapeutic approach for EBVaGC.

## 1. Introduction

The Epstein–Barr virus (EBV), which belongs to the human gamma-herpesvirus family (HHV-4), consist of a ~170-kb double-stranded linear DNA virus [1]. EBV easily infects close family members through salivary transmission during infancy or childhood. Most EBV infections remain asymptomatic, given their ability to establish lifelong latency [2]. EBV is among the most common viruses, with over 95% of world population having been infected and approximately 143,000 deaths having been related to EBV-associated malignancies in 2010 [3]. The EBV virus was the first human tumor virus discovered in Burkitt’s lymphoma cells in 1964 [4]. Thereafter, studies have revealed that EBV infection could cause several different malignancies in lymphoid and epithelial cells. Each EBV-associated malignancy has a unique type of EBV latency phase that exhibits a distinctive pattern of EBV gene expression [5]. Infectious mononucleosis and lymphoproliferative disorder both occur during EBV latency phase III, whereas Burkitt’s lymphoma and gastric carcinoma occur during EBV latency phase I.

Most EBV latent genes are expressed during EBV latency III, whereas only limited viral genes, such as EBERs and EBNA1, are expressed during EBV latency I [6]. Together with other immune evasion strategies, limitations in viral gene expression have been known to help EBV escape the host’s immune system. T cell responses to EBV lytic antigens represent a dominant fraction of EBV-specific T cells generated during primary EBV infection or EBV latency III. Although these EBV-specific T cells aggressively respond to EBV lytic cycle antigens, they barely respond to EBV latent cycle antigens [7]. Moreover, some latent genes, such as *BNLF2a*, *BGLF5*, and *BILF1*, have been reported to interfere with HLA-I antigen presentation from EBV-infected cells. Thus, the switch from latency to lytic infection is a major requisite for the treatment of EBV-associated malignancies [8].

EBV-associated gastric carcinoma (EBVaGC), first discovered in 1992, accounts for 10% of all gastric carcinomas worldwide [9]. This malignancy exhibits male predominance (approximately twice as many as in females), occurs preferably in the proximal stomach or remnant stomach, and has a favorable overall and disease-free survival [10,11]. EBVaGC has been known to feature epigenetic characteristics, such as *PIK3CA* mutation; *CDKN2A* silencing; JAK2, PD-L1, and PD-L2 overexpression; immune cell signaling enrichment; and CpG island methylator phenotype (CIMP) [12]. In particular, EBVaGC has unique hypermethylation phenotypes that allow for higher proportions of DNA methylation than any other gastric cancer [12]. These characteristics help explain why EBVaGC exhibits more distinct phenotypes than EBV-negative gastric carcinoma (EBVnGC). Epigenetic processes, including chromatin remodeling, histone acetylation, and DNA methylation, can regulate gene expression without altering DNA sequences [13,14]. DNA methylation is a well-known epigenetic alteration that occurs on cytosine residues [15] wherein methyl groups (-CH_3_) are added at 5′-carbon of the cytosine ring, subsequently forming 5′-methylcytosine (5^m^C). These methylations frequently occur at cytosine–guanine dinucleotides (CpGs), with condensed CpG regions, called CpG islands. Promoter CpG islands are one of the major regions in which DNA methylation controls gene transcription [16]. Aside from promoter regions, methylation of the untranslated region (UTR), gene body, and exon 1 can also control gene transcription in several cancers [17,18].

Most platinum-based anticancer drugs used as first-line chemotherapeutic treatment for several types of cancer patients are genotoxic [19]. Accordingly, they can selectively bind with genomic DNA and form DNA cross-links within or between DNA strands [20]. DNA cross-linking interferes with DNA replication and transcription and triggers a DNA damage response, leading to cell death [21]. Cisplatin, approved for clinical chemotherapy in 1971 as the first platinum analog, is currently being broadly used as a first-line cancer treatment, alone or in combination with other chemotherapeutics [22]. However, cisplatin-based treatments have shown limitations in some patients owing to side effects, including nephrotoxicity, lack of therapeutic effects, and development of tumor resistance to cisplatin [20]. For instance, one study showed that metastatic nasopharyngeal carcinoma (NPC) usually developed resistance after six cycles of cisplatin-based chemotherapy [23]. Numerous molecular mechanisms have been suggested to have promoted resistance in the NPC cases. Another study showed that EBV latency modulates the *p53* gene to produce chemoresistance in EBV-carrying cells [24]. Dysregulated epigenetic machineries are able to disturb the normal expression of tumor-suppressor genes and oncogenes, resulting in tumorigenesis [25].

DNA methylation is one of epigenetic alterations associated with tumorigenesis. EBV infection clearly affects the DNA methylation that subsequently leads to the development of different cancers. For example, DNA hypermethylation of tumor-related genes is more frequent in EBV-positive Hodgkin’s lymphoma (HL) cases than EBV-negative HL cases [26]. This hypermethylation is observed in NPC and EBVaGC as well [27,28], suggesting the distinct role of EBV in DNA methylation for tumorigenesis. Other exemplary studies have shown that alterations in DNA methylation patterns are strongly associated with not only disease prognosis but also patient survival after anticancer therapy [29]. The altered DNA methylation patterns have been used as key biomarkers for predicting disease prognosis and the efficacy of anticancer therapy [30].

Thus, we hypothesized that EBVaGC with a hypermethylated DNA phenotype would have quite different therapeutic responses to anticancer drugs compared to EBVnGC. In support of this hypothesis, one study showed that EBVaGC was more resistant to docetaxel and 5-fluorouracil-based chemotherapies than EBVnGC [31]. However, differences in responses to platinum-based chemotherapy between EBVaGC and EBVnGC still remain unclear due to a lack of presenting molecular mechanisms. The current study therefore (1) evaluated the bioactive effects of a combination of cisplatin and 5-Azacytidine (5-AZA) on EBV and EBVaGC, (2) determined the epigenetic mechanisms used by the drug combination to overcome tumor resistance, and (3) presented a novel therapeutic approach for EBVaGC.

## 2. Results

### 2.1. EBV Regulates DNMT3A Expression by Cisplatin

**Figure 1:**
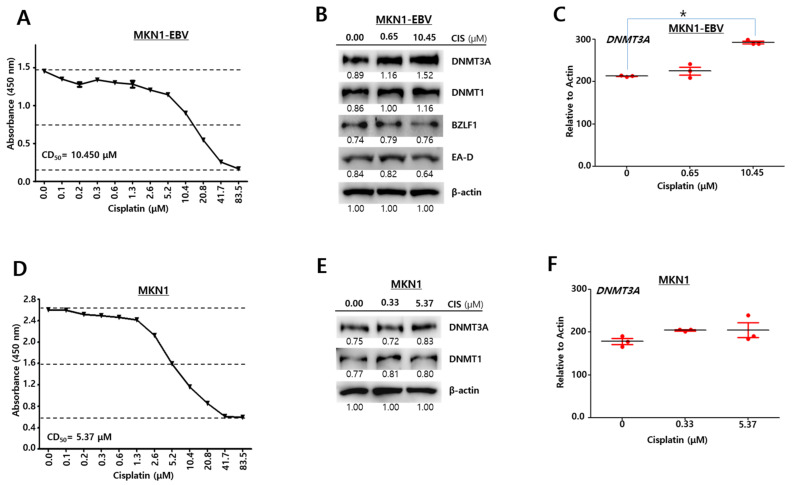
EBV infection regulates *DNMT3A* expression through cisplatin. (**A**) Cytotoxic test of cisplatin against MKN1–EBV cells using CCK-8. (**B**) Western blot assays to investigate the expression patterns of EBV lytic genes (*BZLF1*, *EA-D*) and *DNMT3A* in cisplatin-treated MKN1–EBV cells (48 h treatment). β-Actin was used as a loading control. Band intensities were analyzed using Image J software. (**C**) RT-qPCR assays to quantify relative amounts of *DNMT3A* transcripts in cisplatin-treated MKN1–EBV cells. Statistical significance was evaluated using one-way ANOVA Dunnett’s test (* *p* < 0.05). (**D**) Cytotoxic test of cisplatin against MKN1 cells using CCK-8. (**E**) Western blot assays to investigate expression patterns of EBV lytic genes and *DNMT3A* in cisplatin-treated MKN1 cells. β-Actin was used as a loading control. Band intensities were analyzed using Image J software. (**F**) RT-qPCR assays to quantify relative amounts of *DNMT3A* transcripts in the cisplatin-treated MKN1 cells. Statistical significance was evaluated using one-way ANOVA Dunnett’s test (* *p* < 0.05).

Similar to previous reports, this study also hypothesized that EBV infection affects DNA methylation by regulating DNA methyltransferase (DNMT) and contributes to producing chemo-resistance in EBVaGC. To test this hypothesis, we investigated how EBV infection is associated with the expression of the *DNMT3A* gene in gastric cancer cells. MKN1 cells, which are an EBV-negative gastric cancer cell, were transfected with recombinant EBV bacmid and selected with hygromycin to make MKN1–EBV cells. We first determined that the CD_50_ of MKN1–EBV cells against cisplatin was 10.45 μM (Figure 1A). This CD_50_ was applied to the following experiments with cisplatin-treated MKN1–EBV cells. A Western blot analysis was conducted to investigate the expression pattern of *DNMT3A* proteins in MKN1–EBV cells with cisplatin treatment. We observed the amount of *DNMT3A* proteins increased in proportion to the cisplatin dose (Figure 1B). Cisplatin increased *DNMT3A* protein but decreased *BZLF1* protein and *EA-D* protein in the 48 h post cisplatin treatment. An RT-qPCR assay was carried out to define the expression pattern of *DNMT3A* transcripts in the cisplatin-treated MKN1–EBV cells. Unexpectedly, the amount of *DNMT3A* transcript was significantly reduced in proportion to the cisplatin dose (Figure 1C). We concluded from these results that DNMT3A expression was regulated by cisplatin treatment. To further investigate if EBV infection is associated with the regulation of *DNMT3A* expression by cisplatin, we assessed whether cisplatin regulates DNMT3A expression in MKN1 cells that lack any EBV infection. To this end, we first determined that the CD_50_ of MKN1 cells against cisplatin was 5.37 μM (Figure 1D). This CD_50_ was applied to the following experiments with cisplatin-treated MKN1 cells. A Western blot assay and RT-qPCR assay were conducted to define the expression patterns of *DNMT3A* proteins and transcripts in MKN1 cells with cisplatin treatment. The amount of *DNMT3A* protein and transcript was not significantly changed in the cisplatin-treated MKN1 cells (Figure 1E,F). Taken together, these results suggest that EBV infection is highly associated with *DNMT3A* expression in gastric cancer cells, indicating epigenetic modification.

### 2.2. Cisplatin Upregulates DNMT3A in SNU719 Cells

**Figure 2:**
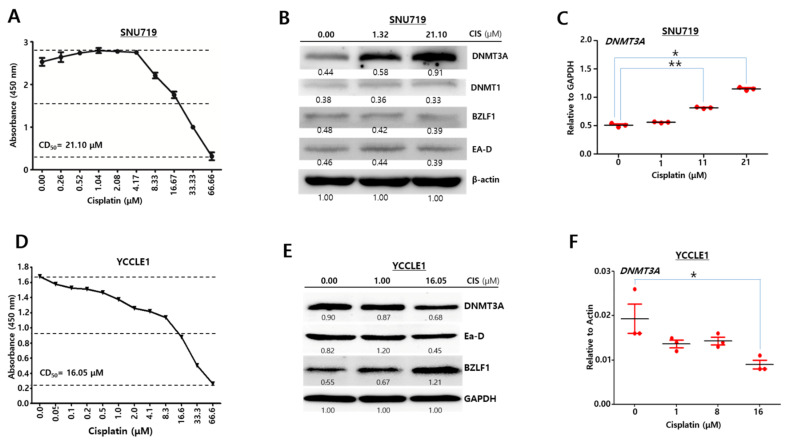
Cisplatin upregulates DNMT3A in SNU719 cells. (**A**) Cytotoxic test of cisplatin against SNU719 cells using CCK-8. (**B**) Western blot assays to investigate expression patterns of EBV lytic gene proteins and DNMT3A proteins in cisplatin-treated SNU719 cells. β-Actin was used as a loading control. Band intensities were analyzed using Image J software. (**C**) RT-qPCR assays to quantify relative amounts of DNMT3A transcript in the cisplatin-treated SNU719 cells. Statistical significance was evaluated using one-way ANOVA Dunnett’s test (*^,^ ** *p* < 0.05). (**D**) Cytotoxic test of cis-platin against YCCLE1 cells using CCK-8. (**E**) Western blot assays to investigate expression patterns of EBV lytic gene proteins and DNMT3A proteins in cisplatin-treated YCCLE1 cells. GAPDH was used as a loading control. Band intensities were analyzed using Image J software. (**F**) RT-qPCR assays to quantify relative amounts of DNMT3A transcripts in cisplatin-treated YCCLE1 cells. Statistical signify cance was evaluated using one-way ANOVA Dunnett’s test (* *p* < 0.05).

Since EBV infection regulated a conditional expression of *DNMT3A* protein in MKN1 cells by cisplatin, we questioned if cisplatin treatment affects *DNMT3A* expression in EBVaGC derived clinically. SNU719 cells and YCCLE1 cells were used exemplarily as EBVaGC in this study. First, we investigated whether cisplatin affects DNMT3A expression in SNU719 cells. The CD_50_ of SNU719 cells against cisplatin was defined as 21.10 µM (Figure 2A). This CD_50_ was applied to following experiments with cisplatin-treated SNU719 cells. Western blot analysis was conducted to investigate the expression pattern of *DNMT3A* proteins in SNU719 cells with cisplatin treatment. We observed that the amount of *DNMT3A* proteins distinguishably increased in proportion to the cisplatin dose. DNMT3A catalyzes 5-methylcytosine methylation, leading to EBV genome methylation (Figure 2B). The activation of most early lytic EBV promoters is inhibited by this genomic methylation [32]. In our study, EBV immediate-early lytic gene, BZLF1 and EBV early lytic gene, EA-D were used to indicate EBV lytic reactivation [33]. In contrast to DNMT3A, BZLF1 and EA-D were slightly reduced in cisplatin-treated SNU719 cells (Figure 2B).

An RT-qPCR assay was also carried out to define the expression pattern of *DNMT3A* transcripts in cisplatin-treated SNU719 cells. We observed that the quantity of *DNMT3A* transcripts was significantly increased in proportion to the cisplatin dose (Figure 2C). These results indicate that cisplatin highly induces to overexpress both *DNMT3A* protein and transcript in SNU719 cells. Secondly, we investigated whether cisplatin affects DNMT3A expression in YCCLE1 cells. The CD_50_ of YCCLE1 cells against cisplatin was defined as 16.05 μM (Figure 2D). This CD_50_ was applied to the following experiments with cisplatin-treated YCCLE1 cells. A Western blot analysis was conducted to investigate the expression pattern of *DNMT3A* proteins in YCCLE1 cells with cisplatin treatment. We observed that the amount of *DNMT3A* proteins was unexpectedly reduced in proportion to the cisplatin dose while the amount of *BZLF1* proteins was increased in cisplatin-treated SNU719 cells (Figure 2E). An RT-qPCR assay was also carried out to define the expression pattern of *DNMT3A* transcripts in cisplatin-treated YCCLE1 cells. We observed that the amount of *DNMT3A* transcripts significantly decreased in high concentration cisplatin (Figure 2F). These results indicate that cisplatin slightly reduces the amount of both *DNMT3A* proteins and transcripts in YCCLE1 cells. Taken together, all results suggest that cisplatin is likely to regulate DNMT3A expression independently of the type of EBVaGC.

### 2.3. Cisplatin Stabilizes DNMT3A Protein

**Figure 3:**
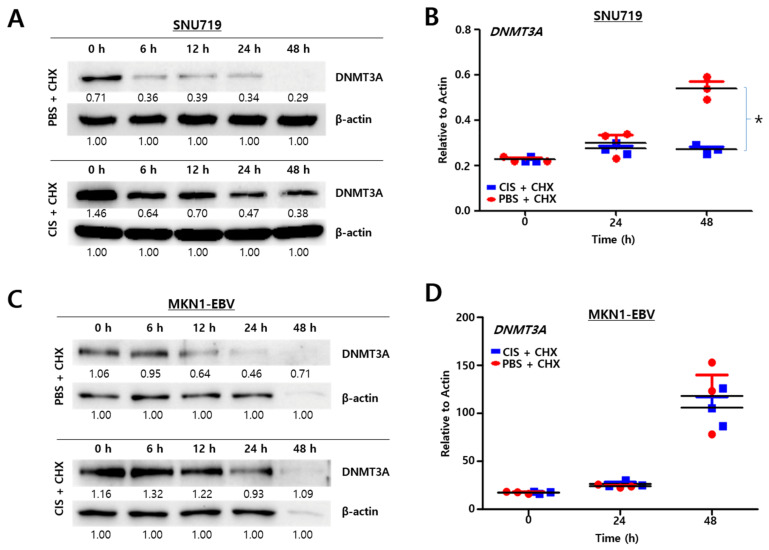
Cisplatin stabilizes *DNMT3A* protein. (**A**) Western blot-based test for the stability of *DNMT3A* protein in SNU719 cells by treating with either cycloheximide (CHX, 100 μg/mL) alone or in combination with cisplatin (CIS, 21.10 μM). β-Actin was used as a loading control. Band intensities were analyzed using Image J software. (**B**) RT-qPCR assay to investigate a change in the quantity of *DNMT3A* transcripts in SNU719 cells by cycloheximide (CHX, 100 μg/mL) treatment. Statistical significance was evaluated using Student’s *t*-test (* *p* < 0.05). (**C**) Western blot-based test for the stability of *DNMT3A* proteins in MKN1–EBV cells by treating with either cycloheximide (CHX, 100 μg/mL) alone or in combination with cisplatin (CIS, 10.45 μM). β-Actin was used as a loading control. Band intensities were analyzed using Image J software. (**D**) RT-qPCR assay to investigate a change in the quantity of *DNMT3A* transcripts in MKN1–EBV cells by cycloheximide (CHX, 100 μg/mL) treatment. Statistical significance was evaluated using Student’s *t*-test (* *p* < 0.05).

Since cisplatin upregulates *DNMT3A* gene in SNU719 cells, we questioned how cisplatin enhanced the amount of *DNTM3A* proteins in SNU719 cells. To this end, it was conducted a protein stability assay to determine cisplatin-mediated fluctuations in DNMT3A stability (Figure 3A). SNU719 cells were treated with cycloheximide (CHX) to block protein synthesis without cisplatin treatment. The amount of *DNMT3A* proteins was decreased considerably 6 h after CHX treatment, with no *DNMT3A* proteins being detected after 48 h. Subsequently, SNU719 cells were cotreated CHX with 21.10 μM of cisplatin at various time points. The amount of *DNMT3A* proteins did not severely decrease considerably 6 h after CHX treatment, compared to the CHX monotreatment. Cotreatment with cisplatin and CHX appeared to reduce the amount of *DNMT3A* proteins and could maintain a detectable amount of *DNMT3A* proteins even after 48 h. In addition, we conducted RT-qPCR assay to investigate if both cisplatin/CHX and PBS/CHX treatments make a difference in producing *DNMT3A* transcripts in SNU719 cells (Figure 3B). The amount of *DNMT3A* transcripts was not different until 24 h post-treatment between cisplatin/CHX and PBS/CHX treatments, while the amount of *DNMT3A* transcripts was significantly different later 48 h post-treatment. Similar to SNU719 cells, we conducted a protein stability assay to determine DNMT3A stability in MKN1–EBV cells while cisplatin was treated (Figure 3C). MKN1–EBV cells were treated with CHX to block protein synthesis without cisplatin treatment. The amount of *DNMT3A* proteins was decreased considerably 6 h after CHX treatment, with no *DNMT3A* proteins being detected after 48 h. Subsequently, MKN1-EBV cells were cotreated CHX with 10.45 μM of cisplatin at various time points. The amount of *DNMT3A* proteins did not severely decrease considerably 6 h after CHX treatment, compared to the CHX monotreatment. Cotreatment with cisplatin and CHX appeared to reduce the amount of *DNMT3A* proteins and could maintain a detectable amount of *DNMT3A* proteins even after 48 h. In addition, we conducted RT-qPCR assay to investigate if both cisplatin/CHX and PBS/CHX treatments make a difference in producing *DNMT3A* transcripts in MKN1–EBV cells (Figure 3D). The amount of *DNMT3A* transcripts was not significantly different until 48 h post-treatment between cisplatin/CHX and PBS/CHX treatments. Taken together, all results indicate that cisplatin stabilizes *DNMT3A* protein in SNU719 cells.

### 2.4. DNMT3A Suppresses Cisplatin-Mediated EBV Lytic Reactivation

**Figure 4:**
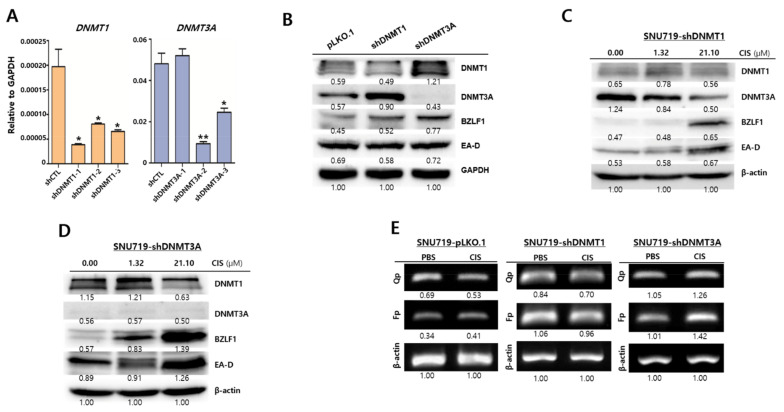
DNMT3A suppresses cisplatin-mediated EBV lytic reactivation. (**A**) qRT-PCR assay to quantify relative amounts of DNMT1 and DNMT3A transcripts in the DNMT1 or DNMT3A knockdown SNU719 cells. These knockdown cells were named SNU719-pLKO.1, SNU719-shDNMT1, and SNU719-shDNMT3A cells. (**B**) Western blot assay to evaluate expression patterns of DNMTs and EBV lytic proteins in SNU719-pLKO.1, SNU719-shDNMT1, and SNU719-shDNMT3A cells. GAPDH was used as a loading control. Band intensities were analyzed using Image J software. (**C**) Western blot assay to investigate expression patterns of DNMTs and EBV lytic proteins in SNU719-shDNMT1 cells which were treated with cisplatin for 48 h. β-Actin was used as a loading control. Band intensities were analyzed using Image J software. (**D**) Western blot assay to investigate expression levels of DNMTs and EBV lytic proteins in SNU719-shDNMT3A cells which were treated cisplatin for 48 h. β-Actin was used as a loading control. Band intensities were analyzed using Image J software. (**E**) RT-semiquantitative (sq) PCR assay to quantify relative transcriptional activities of EBV promoters in SNU719-pLKO.1, SNU719-shDNMT1, and SNU719-shDNMT3A cells. These cells were treated 1.32 μM cisplatin for 48 h. GAPDH and β-actin were used as an internal control. Band intensities were analyzed using Image J software. * *p* < 0.05, ** *p* < 0.01 (Student’s *t*-test).

Given that cisplatin could simultaneously upregulate *DNMT3A* and EBV lytic genes, we determined whether *DNMT3A* protein was required to induce EBV lytic reactivation during cisplatin treatment. To this end, SNU719 cells whose DNMTs were stably knocked down via different shRNAs for DNMT1 and DNMT3A were established. Through qRT-PCR assays, *DNMT1* or *DNMT3A* transcript levels were confirmed to determine DNMT knockdown efficiencies in SNU719 cells (Figure 4A). SUN719 cells produced a remarkably lower quantity of *DNMT1* transcripts than *DNMT3A* transcripts. In spite of that low *DNMT1* transcription, we used both shDNMT1-1 and shDNMT3A-2 to select SNU719-shDNMT1 cells and SNU719-shDNMT3A cells, respectively, for subsequent experiments. Western blot assays were conducted to further confirm DNMT knockdown efficiencies and expression patterns of target genes in DNMT knockdown SNU719 cells (Figure 4B). *DNMT3A* protein was completely knocked down while *DNMT1* was knocked down in slice in SNU719-shDNMT3A cells. Interestingly, EBV lytic *BZLF1* protein was overexpressed in both *DNMT3A* and *DNMT1* knockdown cells compared to pLKO.1 control cells (Figure 4B). Thereafter, we used cisplatin to treat SNU719 cells with either DNMT1 or DNMT3A knockdown. In SNU719-shDNMT1 cells, cisplatin decreased *DNMT3A* protein while increasing both *BZLF1* and *EA-D* proteins (Figure 4C). Similarly, in SNU719-shDNMT3A cells, cisplatin decreased *DNMT1* protein while greatly increasing both *BZLF1* and *EA-D* proteins (Figure 4D).

Given that the depletion of *DNMT3A* proteins could strengthen EBV lytic gene expression, we conducted EBV promoter usage assays to determine whether EBV promoter activities relate to cisplatin-mediated EBV lytic reactivation. Cisplatin treatment in SNU719 cells followed by EBV promoter activity determination showed that SNU719-pLKO.1 and SNU719-shDNMT1 cells did not increase EBV Fp (lytic gene) and Qp (latency gene) promoter activities, whereas SNU719-DNMT3A cells remarkably enhanced Fp and Qp promoter activities (Figure 4E). Consistent with previous results, our results showed that cisplatin preferred DNMT3A loss over DNMT1 loss for EBV lytic reactivation through the activation of EBV lytic promoters. Consistent with previous data, all these results suggest that cisplatin likely upregulated EBV lytic genes in the absence of *DNMT3A* proteins.

### 2.5. Depletion of DNMT3A Proteins Accelerates Cisplatin-Mediated Cell Death

**Figure 5:**
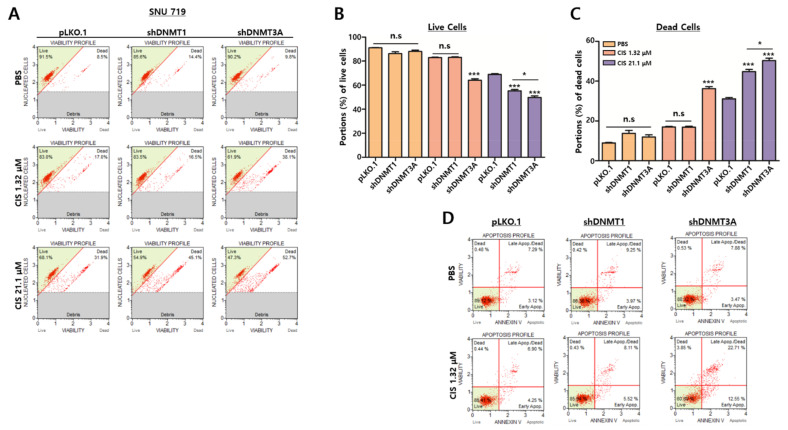
Depletion of *DNMT3A* proteins accelerates cisplatin-mediated cell death. (**A**) Representative flow cytometry dot plots showing viabilities of SNU719-pLKO.1, SNU719-shDNMT1, and SNU719-shDNMT3A cells. These cells were treated with both 1.32 μM and 21.1 μM cisplatin for 48 h. (**B**,**C**) Quantifications of live and dead cells of SNU719-pLKO.1, SNU719-shDNMT1, and SNU719-shDNMT3A. These cells were treated with both 1.32 μM and 21.1 μM cisplatin for 48 h. Statistical significance was evaluated using Student’s *t*-test (* *p* < 0.05). (**D**) Representative flow cytometry dot plots showing apoptosis profiles of SNU719-pLKO.1, SNU719-shDNMT1, and SNU719-shDNMT3A cells. These cells were treated with both 1.32 μM and 21.1 μM cisplatin for 48 h. n.s.: not significant, * *p* < 0.05, *** *p* < 0.001.

Given that EBV lytic reactivation lyses EBV-infected cells due to an excess of viral DNA replication, we investigated whether the absence of *DNMT3A* proteins could facilitate cisplatin-mediated EBV lytic reactivation by conducting MUSE cell count and viability assays to measure SNU719 cell viability after cisplatin treatment. At cisplatin concentrations of 1.32 and 21.1 μM, no significant difference in viability was observed between SNU719-shDNMT1 cells, compared to SNU719-pLKO.1 cells. On the other hand, cisplatin appeared significantly cytotoxic to SNU719-shDNMT3A cells compared to others (Figure 5A). When we quantified portions of live cells (Figure 5B) and dead cells (Figure 5C), the portions of SNU719-shDNMT3A cells were significantly smaller or larger than those of SNU719-shDNMT1 cells and SNU719-pLKO.1 cells.

Most EBV lytic reactivations have been known to be followed by apoptotic cell death [34]. We also observed that the depletion of *DNMT3A* proteins enhanced cisplatin cytotoxicity. Thus, we subsequently conducted a MUSE annexin V and dead cell assay to determine whether cisplatin treatment can lead to SNU719 apoptotic death. Accordingly, 1.32 μM of cisplatin promoted no active apoptotic cell death in SNU719-shDNMT1 cells but did so in SNU719-shDNMT3A cells (Figure 5D). These results indicate that apoptosis is one of the main factors that enhance the cisplatin susceptibility of SNU719-shDNMT3A cells.

### 2.6. Methylation Occurs on ATM Transcriptional Control Region

**Figure 6:**
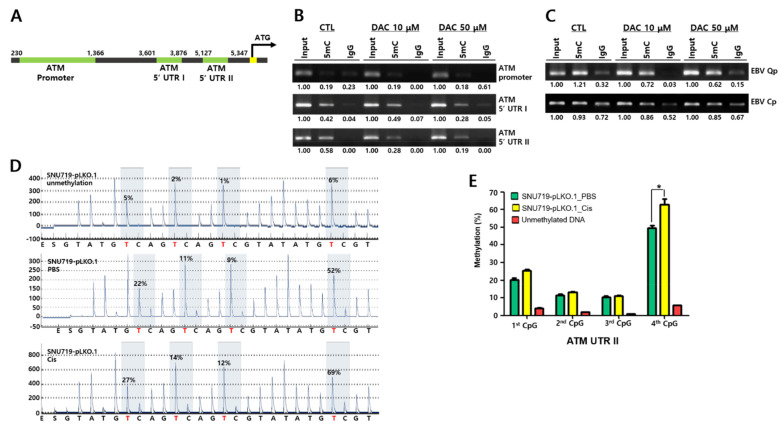
Methylation occurs on ATM transcriptional control region. (**A**) Map of 5′-UTR of ATM gene. The ATM transcriptional control region is composed of the ATM promoter, ATM 5′-UTR I, and ATM 5′-UTR II. (**B**) Methylated DNA im-immunoprecipitation (MeDIP) assay to evaluate DNA methylation in the ATM transcriptional control region of SNU719 cells treated with 2′-deoxy-5′-azacytidine (DAC, 10 or 50 μM, 72 h). (**C**) MeDIP assay to evaluate DNA methylation patterns in the EBV promoter Qp and Cp of SNU719 cells treated with 2′-deoxy-5′-azacytidine (DAC, 10 or 50 μM, 72 h). (**D**) Pyrosequencing profile of CpG methylation (%) in ATM 5′-UTR II of SNU719-pLKO.1 (CTL) and SNU719-shDNMT3A cells treated with cisplatin (1.32 μM, 48 h). (**E**) Quantification of CpG methylation (%) in ATM 5′-UTR II of SNU719- pLKO.1 cells treated with cisplatin (1.32 μM, 48 h). * *p* < 0.05 (Student’s *t*-test).

Saha et al. reported that the EBV infection of naïve B lymphocytes leads to global transcriptional repression of several genes associated with the cell cycle and apoptosis, as well as DNA damage repair, and other tumor suppressor genes [35]. Previous studies have showed that cisplatin induces apoptosis through DNA damage response signaling [36]. ATM is a major sensor enzyme for DNA damage recognition and determines apoptosis and cell cycle arrest. Thus, we sought to examine whether DNA methylation could regulate ATM expression. To this end, we first investigated the *ATM* genomic locus and identified the *ATM* promoter region and two *ATM* 5′UTRs (Figure 6A).

Secondly, we applied the *ATM* locus to analyze the methylation pattern of *ATM* (NC_000011.10) using the Sequence Manipulation Suite (https://www.bioinformatics.org/sms2/cpg_islands.html (accessed on 03, July, 2019) and MEXPRESS websites (https://mexpress.be/ (accessed on 03, July, 2019) [37]. Our results showed that *ATM* promoter region contained several CpG islands (108,222,695–108,222,969) and 5′-UTR I and II regions (108,225,427–108,225,760 and 108,226,925–108,227,273), as well as over 50% CpG dinucleotides (Supplemental Figure S1).

Thirdly, due to its plentiful CpG dinucleotides, we conducted MeDIP-PCR and pyrosequencing assays to analyze the methylation of the ATM control region using DNA methylation inhibitor DAC, a trigger for proteosomal degradation of the DNA methyltransferase family [34]. We first treated SNU719 cells with 10 and 50 μM of DAC for 72 h and isolated gDNA from SNU719 cells to perform a MeDIP assay with anti-5′-methylcytosine antibody. The resultant immune-precipitated DNA samples were subjected to MeDIP-PCR assay and pyrosequencing assay. First, a MeDIP-PCR assay was conducted to investigate DNA methylation on the *ATM* transcriptional control region (Figure 6B). DAC-treated SNU719 cells could strongly and dose-dependently induce demethylation on the *ATM* 5′-UTR II, while the cells did not change the DNA methylation in the *ATM* promoter and *ATM* 5′-UTR I. Secondly, we investigated DNA methylation on the EBV promoter Qp and Cp regions (Figure 6C). DAC-treated SNU719 cells could strongly induce demethylation on the EBV Qp promoter but not Cp promoter.

Fourthly, we further quantitatively analyzed methylation patterns in the ATM 5′-UTR II region using bisulfite modification and pyrosequencing analysis (Figure 6D). As the ATM 5′-UTR II region contained several CpG bases, we could clearly detect DNA methylation in the region of SNU719 cells treated with cisplatin (21.1 μM, 48 h). The fourth CpG dinucleotide on the ATM 5′-UTR II region was hypermethylated in SNU719 cells. This hypermethylation was increased further by cisplatin treatment (69%) than by PBS treatment (52%) (Figure 6D). Three independent experiments were carried out to draw out the statistical significance of the methylation difference of SNU719-pLKO.1 cells treated with PBS or cisplatin (Figure 6E). Cisplatin treatment could induce more DNA methylation on the *ATM* 5′-UTR II than PBS treatment.

### 2.7. Depletion of DNMT3A Proteins Upregulates ATM through Demethylation

**Figure 7:**
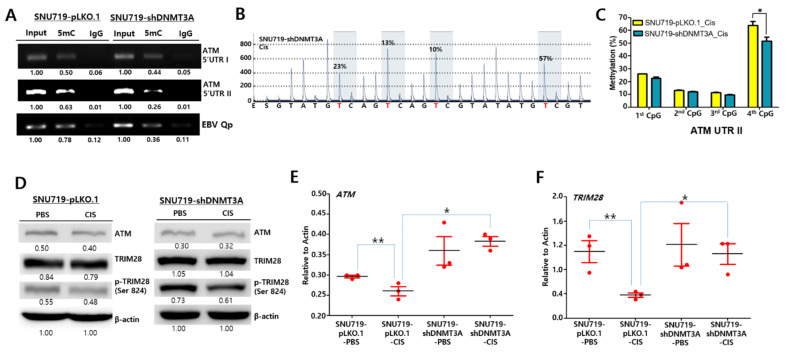
Depletion of DNMT3A proteins upregulates ATM through demethylation. (**A**) MeDIP assay to determine DNA methylation patterns in ATM 5′-UTR in SNU719-pLKO.1 and SNU719-shDNMT3A cells, respectively. (**B**) Pyrosequencing profile of CpG methylation (%) in ATM 5′-UTR II in SNU719-shDNMT3A cells treated with cisplatin (1.32 μM, 48 h). (**C**) Quantification of CpG methylation (%) in ATM 5′-UTR II of SNU719-pLKO.1 and SNU719-shDNMT3A cells. These cells were treated with cisplatin (1.32 μM, 48 h). * *p* < 0.05 (Student’s *t*-test). (**D**) Western blot assay to evaluate the expression patterns of ATM proteins, TRIM28 proteins, and pTRIM28 proteins in SNU719-pLKO.1 and SNU719-shDNMT3A cells. These cells were treated with 1.32 μM cisplatin for 48 h. β-Actin was used as a loading control. Band intensities were analyzed using Image J software. (**E**,**F**) RT-qPCR assays to quantify relative amounts of ATM and TRIM28 transcripts in SNU719-pLKO.1 and SNU719-shDNMT3A cells, respectively. These cells were treated 1.32 μM cisplatin for 48 h. Statistical significance was evaluated using a one-way ANOVA Dunnett’s test (*^,^ ** *p* < 0.05).

Since *ATM* 5′-UTR II was highly susceptible to DNA methylation, we examined the methylation of the 5′-UTR II region in SNU719-shDNMT3A cells using a MeDIP-PCR assay. Accordingly, the loss of *DNMT3A* proteins reduced methylation in the *ATM* 5′-UTR II regions and EBV Qp promoter but not in the *ATM* 5′-UTR I region (Figure 7A).

We further quantitatively analyzed methylation patterns in the *ATM* 5′-UTR II region of SNU719-shDNMT3A cells treated cisplatin (21.1 μM, 48 h). Hypermethylation in the fourth CpG dinucleotide on the *ATM* 5′-UTR II region decreased in SNU719-shDNMT3A cells (57%), compared to SNU719-pLKO.1 cells (69%) (Figure 7B). Cisplatin did not affect DNA hypermethylation in SNU719-shDNMT3A cells. Three independent experiments were carried out to draw out the statistical significance in the methylation difference between SNU719-shDNMT3A cells and SNU719-pLKO.1 cells after cisplatin treatment (Figure 7C).

ATM activation following DNA damage induces self-activation and phosphorylates downstream proteins, including TRIM28 [38]. One exemplary study showed that phosphorylated TRIM28 appeared to be associated with EBV lytic reactivation [39]. Thus, we determined whether cisplatin induces DNA damage response signaling in SNU719 cells (Figure 7D). In SNU719-pLKO.1 and SNU719-shDNMT3A cells, 1.32 μM of cisplatin did not significantly induce TRIM28 phosphorylation at serine 824. However, cisplatin subtly decreased *ATM* protein in SNU719-pLKO.1 cells while subtly increasing *ATM* protein in SNU719-shDNMT3A cells. In addition, we evaluated whether the loss of *DNMT3A* proteins affects the production of *ATM* and *TRIM28* transcripts (Figure 7E,F). Cisplatin significantly decreased the amount of *ATM* and *TRIM28* transcripts in SNU719-pLKO.1 cells. In addition, the loss of *DNMT3A* proteins significantly increased the amount of *ATM* and *TRIM28* transcripts in cisplatin-treated SNU719 cells. These results indicate that the loss of *DNMT3A* protein is required to upregulate *ATM* and *TRIM28* in cisplatin-treated SNU719 cells. Taken together, all results suggest that DNMT3A is likely to regulate ATM-dependent DNA damage signaling in the presence of cisplatin.

### 2.8. Cisplatin Has a Synergistic Anti-EBV Effect with 5-AZA in SNU719 Cells

**Figure 8:**
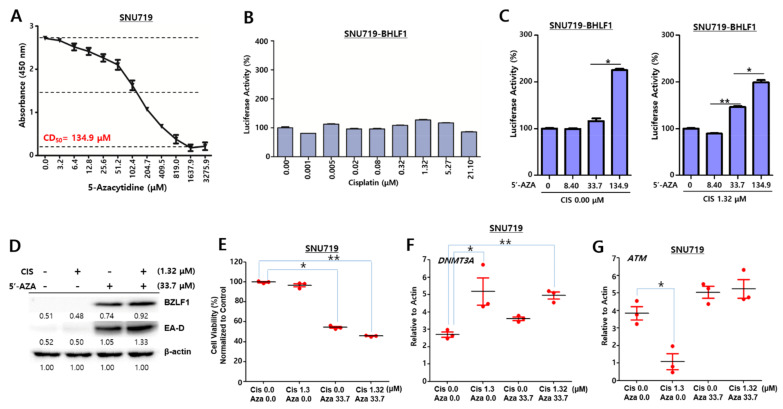
Cisplatin has a synergistic anti-EBV effect with 5-Azacytidine (5-AZA) in SNU719 cells. (**A**) Cytotoxic test of 5-AZA against SNU719 cells using CCK-8. (**B**) Luciferase assay to measure the EBV-BHLF1 promoter activity in SNU719-BHLF1 cells. These cells resulted from SNU719 cells by transfecting a pPL6 plasmid-linking EBV-BHLF1 promoter. SNU719-BHLF1 cells were treated with cisplatin in a serial dilution from CD_50_ (0.00 ~ 21.10 µM). (**C**) Luciferase assay to quantify EBV lytic gene *BHLF1* expression in SNU719-BHLF1 cells. These cells were treated with 5-AZA in combination with cisplatin in dose-dependent manner. Statistical significance was evaluated using Student’s *t*-test (* *p* < 0.05). (**D**) Western blot assay to evaluate expression patterns of EBV lytic gene proteins in SNU719 cells. These cells were treated with either cisplatin (1.32 μM) alone or cisplatin combined with 5-AZA (33.7 μM). (**E**) Cell viability assay to evaluate viability of SNU719 cells. These cells were treated with either cisplatin (1.32 μM) alone or cisplatin combined with 5-AZA (33.7 μM). Statistical significance was evaluated using a one-way ANOVA Dunnett’s test (* *p* < 0.05). (**F**,**G**) RT-qPCR assays to quantify amounts of *DNMT3A* and *ATM* transcripts in SNU719 cells. These cells were treated with either cisplatin (1.32 μM) alone or cisplatin combined with 5-AZA (33.7 μM). Statistical significance was evaluated using a one-way ANOVA Dunnett’s test (*^,^ ** *p* < 0.05).

5-Azacytidine (5-AZA) therapy has been used to treat lymphoma, given its ability to trigger proteosomal degradation of DNA methyltransferases [40]. As such, we determined whether cisplatin combined with 5-AZA could produce synergistic effects in inducing either EBV lytic reactivation or apoptosis. To test this hypothesis, we first evaluated 5-AZA-mediated cytotoxicity in SNU719 cells and subsequently determined a 5-AZA CD_50_ to be 134.9 μM (Figure 8A). Secondly, we investigated whether cisplatin enables the promotion of EBV lytic reactivation in SNU719 cells carrying an EBV *BHLF1* promoter (Figure 8B). A luciferase assay was conducted using SNU719-BHLF1 cells as an indicator of EBV lytic gene expression. Considering that the CD_50_ of SNU719 cells against cisplatin is 21.10 μM, treatment involved various concentrations of cisplatin (0.00–21.10 μM) for 48 h. We observed that all cisplatin concentration groups did not significantly induce EBV lytic gene expression. Thirdly, a SNU719-BHLF1 cell-based luciferase assay was conducted to determine whether the combination of cisplatin and 5-AZA could synergistically induce EBV lytic reactivation (Figure 8C). 5-AZA alone at high concentrations, such as 134.9 μM, was able to induce EBV lytic reactivation. However, 5′-AZA and cisplatin cotreatment was able to induce a greater EBV lytic reactivation compared to a single treatment, in a dose-dependent manner. Moreover, 1.32 and 21.10 μM of cisplatin combined with 33.7 μM of 5-AZA was able to synergistically induce EBV lytic reactivation, indicating that the lowest effective concentration of such a combination was 1.32 μM of cisplatin and 33.7 μM of 5-AZA. Fourthly, a Western blot assay was also conducted to confirm the synergistic effects of both drugs on EBV lytic reactivation (Figure 8D). A 1.32 μM cisplatin monotreatment did not induce the expression of the EBV lytic gene, *BZLF1* and *EA-D*, while 33.7 μM 5-AZA induced the expression of the EBV lytic genes. Accordingly, combined treatment promoted a greater production of EBV lytic proteins BZLF1 and EA-D1 than either monotreatment. Fifthly, we evaluated the cell viability of SNU719 cells that were treated with cisplatin and 5-AZA (Figure 8E). Only cisplatin-treated cells were as viable as the negative control, whereas 5-AZA-treated cells were significantly less viable than the cisplatin monotreated cells. Cells treated with both cisplatin and 5-AZA were less viable than 5-AZA-treated cells (Figure 8E). Finally, we determined mRNA levels of *DNMT3A* and *ATM* in SNU719 cells that had been treated with cisplatin and 5-AZA (Figure 8F,G). SNU719 cells treated with only cisplatin appeared to upregulate *DNMT3A* but downregulate *ATM*. SNU719 cells with either 5-AZA monotreatment or cotreatment did not show any change in mRNA levels of *DNMT3A* and *ATM*. Taken together, these results indicate that the *DNMT3A* upregulation is likely to downregulate *ATM* in SNU719 cells.

### 2.9. Cisplatin and 5-AZA Has a Synergistic Anti-EBV Effect in MKN1–EBV Cells

**Figure 9:**
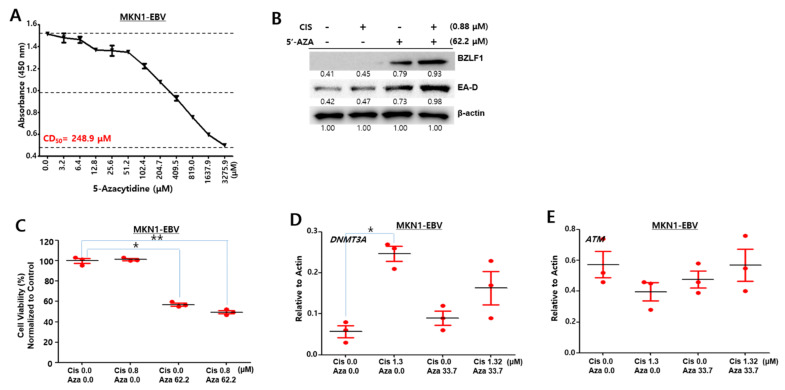
Cisplatin and 5-AZA has a synergistic anti-EBV effect in MKN1–EBV cells. (**A**) Cytotoxic test of 5-AZA against MKN1–EBV cells using CCK-8. (**B**) Western blot assay to evaluate expression patterns of EBV lytic gene proteins in MKN1–EBV cells. These cells were treated with either cisplatin (0.88 μM) alone or cisplatin combined with 5-AZA (62.2 μM). (**C**) Cell viability assay to evaluate viability of MKN1–EBV cells. These cells were treated with either cisplatin (0.88 μM) alone or cisplatin combined with 5-AZA (62.2 μM). Statistical significance was evaluated using a one-way ANOVA Dunnett’s test (* *p* < 0.05). (**D**,**E**) RT-qPCR assays to quantify relative amounts of *DNMT3A* and *ATM* transcripts in MKN1–EBV cells. These cells were treated with either cisplatin (0.88 μM) alone or cisplatin combined with 5-AZA (62.2 μM). Statistical significance was evaluated using a one-way ANOVA Dunnett’s test (*^,^ ** *p* < 0.05).

Since cisplatin combined with 5-AZA could produce synergistic effects in anti-tumor and anti-viral activity against SNU719 cells, we questioned whether the cotreatment produces similar synergistic effects against MKN1–EBV cells. To answer this question, we first evaluated 5-AZA-mediated cytotoxicity in MKN1–EBV cells and subsequently determined a 5-AZA CD_50_ to be 248.9 μM (Figure 9A). Secondly, Western blot assay was also conducted to confirm the synergistic effects of both drugs on EBV lytic reactivation in MKN1–EBV cells (Figure 9B). 0.88 μM Cisplatin monotreatment induced to express *EA-D* gene but *BZLF1* gene, while the cisplatin cotreatment with 62.2 μM 5-AZA induced the expression of both *EA-D* and *BZLF1* genes. Accordingly, combined treatment promoted greater production of *BZLF1* and *EA-D1* proteins than each monotreatment. Thirdly, we evaluated cell viability of MKN1–EBV cells which were treated with cisplatin and 5-AZA therein (Figure 9C). Only cisplatin-treated cells were viable as much as negative control, whereas 5-AZA-treated cells were significantly less viable than the cisplatin monotreated cells. Cells treated with both cisplatin and 5-AZA were further less viable than 5-AZA-treated cells (Figure 9C). Finally, we determined mRNA levels of *DNMT3A* and *ATM* in MKN1–EBV cells which were treated with cisplatin and 5-AZA (Figure 9D,E). MKN1–EBV cells treated with only cisplatin appeared to upregulate *DNMT3A* but downregulate *ATM*. MKN1–EBV cells with either 5-AZA monotreatment or the cotreatment did not show any change in mRNA levels of *DNMT3A* and *ATM*. Taken together, these results also indicate that the *DNMT3A* upregulation is likely to downregulate *ATM* in MKN1–EBV cells.

### 2.10. Depletion of ATM Proteins Reduces Cell Susceptibility to Anticancer Drugs

**Figure 10:**
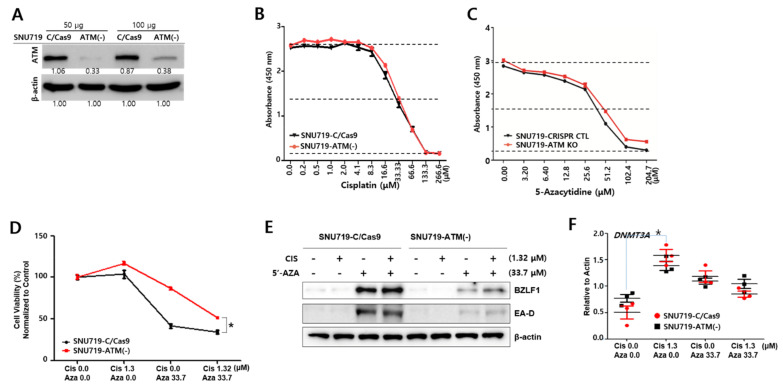
Depletion of *ATM* proteins reduces the susceptibility of SNU719 cells to anticancer drugs. (**A**) Western blot assay to evaluate the efficiency of *ATM* knockdown SNU719 cells. These cells resulted from SNU719 cells after removing *ATM* using CRISPR/Cas9 genomic editing. The resultant cells were named SNU719-C/Cas9 (a negative control) and SNU719-ATM(-)cells. (**B**) Cytotoxic tests of SNU719-C/Cas9 are SNU719-ATM(-) cells against cisplatin using CCK-8. (**C**) Cytotoxic tests of SNU719-C/Cas9 and SNU719-ATM(-) cells against 5′-Azacitidine using CCK-8. (**D**) Cell viability assay to quantify the viability of SNU719-C/Cas9 and SNU719-ATM(-) cells. These cells were treated with either cisplatin (1.32 μM, 48h) alone or in combination with 5-AZA (33.7 μM, 48 h). Statistical significance was evaluated using Student’s *t*-test (* *p* < 0.05). (**E**) Western blot assay to evaluate the expression levels of EBV lytic proteins in SNU719-C/Cas9 and SNU719-ATM(-) cells. These cells were treated with either cisplatin (1.32 μM, 48h) alone or in combination with 5-AZA (33.7 μM, 48 h). (**F**) RT-qPCR assays to quantify amounts of *DNMT3A* transcripts in in SNU719-C/Cas9 and SNU719-ATM(-) cells. These cells were treated with either cisplatin (1.32 μM, 48h) alone or in combination with 5-AZA (33.7 μM, 48 h). Statistical significance was evaluated using a one-way ANOVA Dunnett’s test (* *p* < 0.05).

Our data previously showed that cisplatin treatment enhanced the mRNA levels of *ATM* in the absence of DNMT3A expression. Furthermore, cisplatin treatment decreased *ATM* mRNA while inducing DNMT3A upregulation. In fact, ATM-TRIM28 signaling has been associated with DNA damage response and plays a key role in cisplatin-based chemotherapy and EBV lytic reactivation [41]. Hence, we questioned how ATM affects biological features specific to cisplatin treatment and determined whether the depletion of *ATM* proteins had protective effects against anticancer drugs. To this end, *ATM* knockdown SNU719 cells were first generated using the CRISPR/Cas9 system, their *ATM* knockdown efficiency confirmed, and then named SNU719-ATM(-) cells (Figure 10A).

Secondly, we examined whether the depletion of *ATM* proteins was required for cytotoxicity caused by cisplatin and 5-AZA. CCK8-based cytotoxicity assays showed that SNU719-ATM(-) cells had a higher resistance to both cisplatin and 5-AZA, compared to CRISPR/Cas9 control SNU719 cells, here named SNU719-C/Cas9 cells (Figure 10B,C). Furthermore, we examined the viability of SNU719-ATM(-) cells cotreated with cisplatin and 5-AZA. After 48 h of treatment, SNU719-ATM(-) cells appeared to be significantly more viable than SNU719-C/Cas9 cells at combined drug concentrations (Figure 10D). These results suggest that a functional ATM is required to kill SNU719 cells with anticancer drugs.

Thirdly, we investigated whether ATM is associated with EBV lytic reactivation in the presence of anticancer drugs (Figure 10E). Western blot assay was conducted to test whether SNU719-ATM(-) cells could induce EBV lytic reactivation in the presence of cancer drugs. SNU719-C/Cas9 cells with mono- or cotreatments of cisplatin and 5-AZA clearly induced the EBV lytic genes *BZLF1* and *EA-D*. SNU719-C/Cas9 cells treated with both drugs slightly enhanced EBV lytic proteins compared to either monotreatment. In contrast, SNU719-ATM(-) cells with mono- or cotreatments of cisplatin and 5-AZA expressed a very small quantity of the EBV lytic proteins. These results indicate that functional ATM is essential to induce EBV lytic reactivation by anticancer drugs. Finally, we were curious to ascertain whether *ATM* protein affects *DNMT3A* transcription. Hence, we determined mRNA levels of *DNMT3A* in SNU719-ATM(-) cells with cisplatin and 5-AZA cotreatment (Figure 10F). Expression patterns of *DNMT3A* transcripts were not significantly different in either SNU719-ATM(-) or SNU719-C/Cas9 cells. These results indicate that cisplatin induces the upregulation of the *DNMT3A* gene in advance of the *ATM* gene and thus *DNMT3A* upregulation is not dependent on *ATM* expression. Based on all findings, which show that the depletion of *ATM* proteins reduces the anticancer effects of 5-AZA and cisplatin, it is suggested that ATM is required to achieve full anticancer activity against EBVaGC, SNU719 cells.

### 2.11. A Combination of Cisplatin and 5-AZA Suppresses Tumor Development

**Figure 11:**
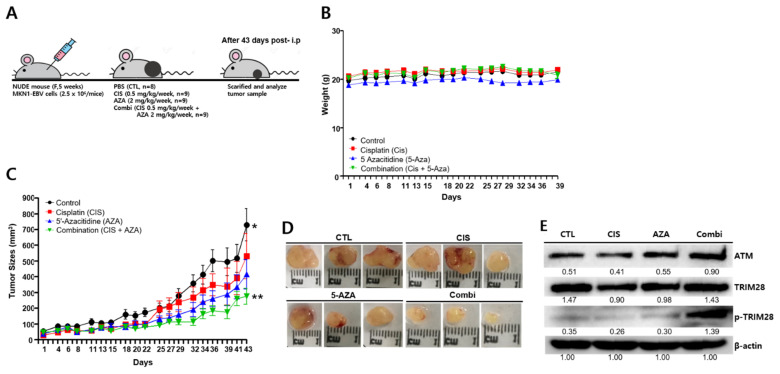
A combination of cisplatin and 5-AZA suppresses tumor development. (**A**) Experimental scheme to evaluate anti-tumor activity using an MKN1–EBV xenograft mouse model. MKN1–EBV xenograft mice were intraperitoneally injected with PBS, cisplatin (0.5 mg/kg), 5-AZA (2 mg/kg), or the same dose of cisplatin and 5-AZA for 43 days. All treatments were intraperitoneally injected twice per week. After that, mice were sacrificed and tumor samples were analyzed. (**B**) Measurement of the body weight of the mice at the time of each injection during the MKN1–EBV xenograft mouse experiment. (**C**) Measurement of tumor sizes of mice at the time of each injection during the MKN1–EBV xenograft mouse experiment. Statistical significance was evaluated using a one-way ANOVA Dunnett’s test (*^,^ ** *p* < 0.05). (**D**) A representative image of MKN1–EBV-derived tumors recovered from xenograft nude mice. (**E**) Western blot assay to evaluate expression levels of ATM, TRIM28, and pTRIM 28 in tumor tissue samples of MKN1–EBV xenograft mice. β-Actin was used as a loading control. Band intensities were analyzed using Image J software.

To determine the anti-tumor effects of combination therapy in vivo, we initially tried to establish a xenograft mouse model with SNU719 cells. However, we could not induce enough tumorigenesis in nude mice by SNU719 cells for further anti-tumor assay (data not shown). Thus, instead of SNU719 cells, we used MKN1–EBV cells to establish a xenograft mouse model (Figure 11A). Like SNU719 cells, MKN1–EBV cells are derived from intestinal-type gastric cancers, but are aggressive enough to form tumors in nude mice [42]. Nude mice were injected subcutaneously with MKN1–EBV cells (5 × 10^6^ cells/mice) and then intraperitoneally injected with PBS, cisplatin (0.5 mg/kg), 5-AZA (2 mg/kg) or a combination of cisplatin and 5-AZA (0.5 mg/kg cisplatin and 2 mg/kg 5-AZA) weekly for 43 days. Xenograft mice were weighed for 39 days following intraperitoneal injection. No change in weight was noted during the xenograft experiment (Figure 11B). Tumor growth was determined in all 35 mice, and tumor volume was measured every 2 days until it reached 1000 mm^3^ (Figure 11C). No difference between each monotreatment group and the combination treatment group was noted for 22 days following intraperitoneal injection. After 25 days of intraperitoneal injection, the cisplatin treatment group showed better suppression of tumor development compared to the combination treatment group. Although 5-AZA repressed tumor development, its effects were suppressed 32 days after intraperitoneal injection. After 43 days of intraperitoneal injection, the combination groups showed significantly suppressed tumor development (Figure 11D).

Next, *ATM* and phosopho-*TRIM28* expression was examined from xenograft tissue samples using a Western blot assay. *ATM* expression was enhanced in the combination treatment groups, while its upregulation was related to TRIM28 phosphorylation (Figure 11E). Consistent with in vitro experiments, the findings presented here suggest that combination therapy exhibited synergistic effects in inhibiting tumor development in an in vivo mouse model.

### 2.12. ATM Is Required to Suppress Tumor Development by Chemotherapy

**Figure 12:**
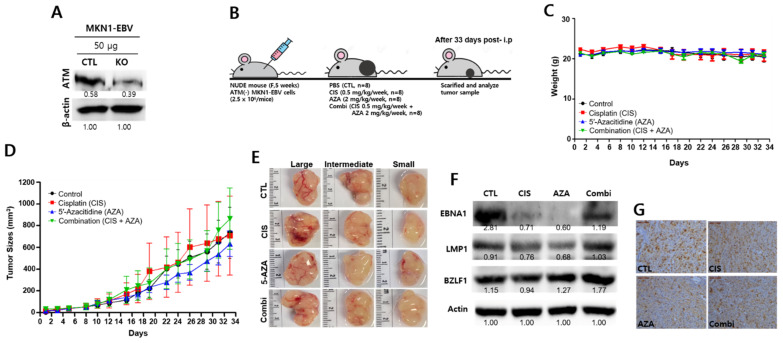
ATM is essential for the suppression of tumor development through chemotherapy. (**A**) Western blot analysis to evaluate the amount of *ATM* proteins in *ATM* knockdown MKN1–EBV cells. These cells resulted from MKN1–EBV cells by removing *ATM* using CRISPR/Cas9 genomic editing. Resultant cells were named MKN1–EBV-C/Cas9 (a negative control) and MKN1–EBV-ATM(-)cells. (**B**) Experimental scheme to evaluate anti-tumor activity using MKN1–EBV-ATM(-) cell xenograft mouse model. Mice were intraperitoneally injected with PBS, cisplatin (0.5 mg/kg), 5-AZA (2 mg/kg), or same dose of cisplatin and 5-AZA for 33 days. (**C**) Measurement of the body weight of the mice at the time of each injection in the MKN1–EBV-ATM(-) cell xenograft mouse experiment. (**D**) Measurement of tumor sizes of mice at the time of each injection in the MKN1–EBV-ATM(-) cell xenograft mouse experiment. (**E**) A representative image of MKN1–EBV-ATM(-) cell derived tumors recovered from xenograft nude mice. Tumors were displayed in accordance with their relative sizes (large, intermediate, small). (**F**) Western blot assay to evaluate expression levels of EBV proteins in MKN1–EBV-ATM(-) cell derived tumor tissue samples. β-Actin was used as a loading control. Band intensities were analyzed using Image J software. (**G**) Representative images of immunohistochemistry (IHC) staining of EBNA1 in sections from the tumor tissues of MKN1–EBV-ATM(-) cell xenograft mice. Scale bar, 100 μm.

Since ATM is required both to kill EBVaGC and induce EBV lytic reactivation in our study, we questioned whether ATM is required to suppress tumor development by chemotherapy. For this purpose, we evaluated the necessity of ATM for anti-tumor effects with a combination of cisplatin and 5-AZA using an in vivo mouse model. *ATM* knockdown MKN1–EBV cells were constructed using CRISPR/Cas9 genomic editing and the *ATM* knockdown efficiency was confirmed. These were named MKN1–EBV-ATM(-) cells (Figure 12A).

Resultant MKN1–EBV-ATM(-) cells were injected subcutaneously into nude mice; 2.5 × 10^6^ cells/mice. Upon developing tiny tumors, nude mice were then intraperitoneally injected weekly for 33 days with the following drugs (Figure 12B): PBS (control), cisplatin (0.5 mg/kg), 5-AZA (2 mg/kg), and a combination of cisplatin and 5-AZA (0.5 mg/kg cisplatin and 2 mg/kg 5-AZA).

Mouse body weight did not change during the xenograft experiment (Figure 12C). We determined tumor growth in all 32 mice by measuring tumor volume every two days until the tumor volume reached 1000 mm^3^ (Figure 12D). No statistical difference between any single treatment group and the combination treatment group was noted for 33 days following intraperitoneal injection. After 33 days of intraperitoneal injection, the combination groups did not show significantly suppressed tumor development (Figure 12E). Subsequently, EBV gene expression was examined from xenograft tissue samples using Western blot assay. Compared to the PBS control group, EBV latent genes were not significantly upregulated in the combination treatment group while the EBV lytic gene *BZLF1* was upregulated in the combination group (Figure 12F). Immunohistochemistry was also conducted to test whether EBV latent infection in the xenograft was maintained even though *ATM* gene was knocked out in the MKN1–EBV xenograft (Figure 12G). DAB (3, 3′-diaminobenzidine)-positive spots (DPSs) indicating EBNA1 staining in this IHC assay were observed in all four xenograft tissues treated with either cisplatin or 5-AZA separately or in combination (Supplemental Figure S2). Interestingly, the number of DPSs per section of the control tissues was not significantly altered compared to the combination-treated tissues. However, the number of DPSs of the control’s tissues was significantly higher than that of the separated cisplatin or 5-AZA tissues. These results indicate that combination therapy requires a functional ATM to exhibit synergistic effects in suppressing tumor development in an in vivo mouse model.

### 2.13. DNMT3A and ATM Are Associated with Overall Survival

**Figure 13:**
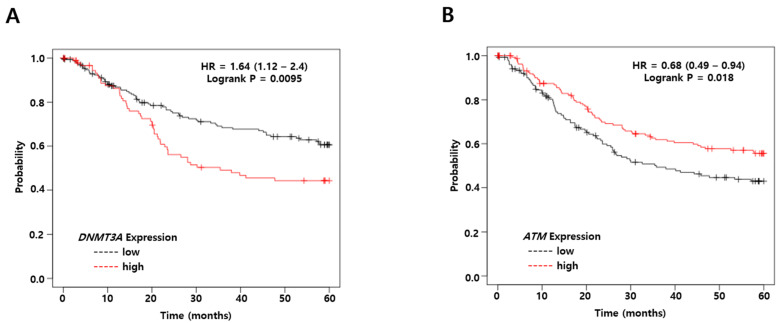
DNMT3A and ATM are associated with overall survival. The 5-year overall survival (OS, *n* = 881) rates were analyzed for gastric cancer cases from the Kaplan–Meier plotter. A total of 320 patients were analyzed from the following datasets: GSE14210, GSE15459, GSE22377, GSE29272, GSE51105, and GSE62254. Input genes were DNMT3A (Affy id: 222640_at) and ATM (Affy id: 212672_at). These analyses were restricted to Lauren classification intestinal (*n* = 336) types of gastric cancers. (**A**) Overall survival curves of DNMT3A expression in patients with gastric carcinoma using the Kaplan–Meier plotter. (**B**) Overall survival curves of ATM expression in patients with gastric carcinoma using the Kaplan–Meier plotter. HR: Hazard ratio.

To evaluate whether *DNMT3A* or *ATM* expression was related to survival rates in patients with gastric carcinoma, the relationship between DNMT3A or ATM and clinical outcomes was analyzed using a Kaplan–Meier plotter http://www.kmplot.com/ (accessed on 7 April 2021) [43]. We were not able to use exclusively EBV-positive gastric cancer patients because the number of cases was too small to get any meaningful result. Thus, we included all gastric cancer patients in our Kaplan–Meier analysis although most cases were EBV-negative gastric cancer patients. Thereafter, we investigated how the mRNA levels of *DNMT3A* and *ATM* were correlated with the overall survival of gastric cancer patients. Kaplan–Meier analysis showed that a low *DNMT3A* mRNA level was significantly correlated with better overall survival from gastric carcinoma. Meanwhile, high *DNMT3A* mRNA levels implied poorer overall survival from gastric carcinoma (*p* = 0.0095, hazard ratio = 1.64; Figure 13A). On the other hand, high *ATM* mRNA levels were associated with better overall survival but low *ATM* mRNA levels implied poorer overall survival from gastric cancer (*p* = 0.018, hazard ratio = 0.68; Figure 13B). Taken together, these findings suggest that both *DNMT3A* downregulation and *ATM* upregulation are likely to produce better overall survival outcomes for gastric cancer patients.

## 3. Discussion

DNA methylation can be an attractive approach to epigenetic modulation favorable for tumor development and related chemotherapeutic resistance [16]. DNA methyltransferases (DNMTs) responsible for DNA methylation have been known to be overexpressed in cancer cells. Moreover, their upregulation has been strongly associated with the loss of tumor suppressor genes (TSG), with the resultant TSG suppression being a good biomarker for a positive prognosis in patients with cancer [29]. In fact, DNA hyper- or hypomethylations have been strongly correlated with chemoresistance and survival rate in gastric carcinoma [44]. Therefore, the biological roles of DNA methylation in the development of EBV-associated cancer should be further understood to establish better therapeutic approaches. The current study determined (1) the anticancer effects of platinum-based anticancer drugs, (2) epigenetic mechanism used for cisplatin-mediated activities, and (3) synergistic anti-EBV effects of cisplatin and 5′-azacytidine.

Firstly, our study showed that EBV infection induces the upregulation of *DNMT3A* depending on types of EBVaGC. EBV infection could upregulate *DNMT3A* in MKN1 cells through cisplatin treatment. Similarly, EBVaGC SNU719 cells highly overexpressed *DNMT3A* with cisplatin while EBVaGC YCCLE1 cells did not. Cisplatin treatment slightly increased *BZLF1* proteins while reducing *DNMT3A* proteins in YCCLE1 cells. We could infer some reasons why these cellular differences occur in responding to cisplatin treatment. A possible reason is related to the difference in the ability to induce EBV lytic reactivation. A conventional TPA treatment was not able to induce the EBV lytic cycle in YCCLE1 cells, but was enough for EBV lytic reactivation in SNU719 cells [45]. There was no detection of BZLF1, BRLF1, BMRF1, or BHRF1 in YCCLE1. However, these proteins were detectable in SNU719 cells. DNA methylation is included in the epigenetic alterations associated with EBV-associated tumorigenesis. In cells with EBV type I latency, the promoters of EBV lytic genes are intensively repressed and suppressed by DNA methylation [46]. Thus, previous studies led to speculation that DNMT3A in YCCLE1 cells might not play a role as a key regulator for expressing EBV lytic genes, which might be associated with a loss of cisplatin-mediated *DNMT3A* upregulation. In contrast, DNMT3A might keep epigenetic control of the expression of EBV lytic genes in SNU719 cells and MKN1–EBV cells. The maintenance of DNMT3A in epigenetic control might contribute to cisplatin-mediated *DNMT3A* upregulation. That is, cisplatin was likely to unsystematically induce EBV lytic reactivation and DNMT3A might be overexpressed to suppress the EBV lytic reactivation. This is a reasonable inference, yet further studies are necessary to have a clear understanding of the loss of DNMT3A upregulation in YCCLE1 cells.

Secondly, our study showed that cisplatin alone could not induce EBV lytic reactivation but did dose-dependently upregulate *DNMT3A*. Interestingly, cisplatin-mediated *DNMT3A* upregulation had adverse effects on the expression of EBV lytic genes in SNU719 cells. Moreover, cisplatin was able to stabilize *DNMT3A* proteins from proteasome degradation in SNU719 cells. These results imply that DNMTs were related to the cytotoxicity of cisplatin on SNU719 cells. After further investigating the role of DNMT1 or DNMT3A on EBV lytic reactivation, we found that knockdown of either DNMT1 or DNMT3A altered EBV lytic reactivation in SNU719 cells. In particular, cisplatin treatment greatly upregulated the EBV immediate-early (IE) lytic gene, BZLF1, and accelerated apoptotic cell death in SNU719-shDNMT3A cells. These results suggest that DNMT3A plays a key role in suppressing EBV lytic reactivation through de novo methylation. Consistently, several clinical studies have reported that DNA hypermethylation occurred more frequently in EBVaGC than in EBVnGC [44,47]. Moreover, EBV infection in B cells has been shown to cause DNA hypermethylation in host genes responsible for the cell cycle, DNA damage repair, and apoptosis [35]. Therefore, we can reasonably infer a mutual regulation between DNMT3A and EBV lytic reactivation.

Thirdly, we determined how DNMT3A plays a key role in controlling the EBV lifecycle. Based on several previous studies, ATM kinase activity was selected for further investigation, given that the ATM kinase orchestrates the DNA damage response and induces EBV lytic reactivation in EBV-infected B cells and LCLs [48]. Interestingly, we observed that *ATM* protein and mRNA were considerably upregulated in the absence of DNMT3A, while the 5′-UTR of *ATM* was significantly less methylated after DAC treatment. These findings implied that DNMT3A likely hypermethylated the *ATM* 5′-UTR, with the resultant *ATM* downregulation possibly preventing both a DNA damage response and EBV lytic reactivation. Consistent with our results, studies have shown that the DNA damage response could be considered a cellular stress similar to hypoxia and that differentiation could effectively turn on EBV lytic reactivation in epithelial cells [33]. ATM has been considered one of the key transducers for the DNA damage response [49] and plays an important role in the signaling pathways commonly activated during EBV lytic reactivation [41,50]. Moreover, the *BGLF4* protein, one of EBV’s early lytic proteins, had previously been shown to phosphorylate topoisomerase II and TIP60 to activate the DNA damage response [51]. Therefore, EBV lytic reactivation is closely linked to DNA damage response.

Fourthly, the current study further investigated how the ATM signaling pathway was involved in EBV lytic reactivation. Previous studies have reported that chloroquine induced EBV lytic reactivation by activating TRIM28 phosphorylation at serine 824 in Burkitt’s lymphoma cells, with the resultant ATM-dependent TRIM28 phosphorylation being required for the upregulation of *BZLF1* and *EA-D* in Burkitt’s lymphoma and lymphoblastoid cells [41]. Aside from EBV, human cytomegalovirus has also been shown to require ATM-dependent TRIM28 phosphorylation for the biological switch from the latency to the lytic phase [52]. Thus, we determined whether cisplatin phosphorylated TRIM28 and whether the resultant phosphorylation was involved in ATM-mediated EBV lytic reactivation. Accordingly, our results showed that cisplatin treatment in the absence of DNMT3A facilitated (1) *ATM* upregulation, (2) ATM-dependent TRIM28 phosphorylation, and (3) EBV lytic reactivation. Furthermore, our study demonstrated that *ATM* knockdown SNU719 cells found cisplatin less cytotoxic than control cells, suggesting the disabling of EBV lytic reactivation due to the loss of ATM kinase activity. Consistent with previous studies, all data presented herein support the notion that the ATM-TRIM28 pathway plays a key role in suppressing EBV lytic reactivation during cisplatin treatment.

Finally, in vivo experiments were conducted to confirm the in vitro synergistic effect of the anticancer drug and epigenetic modulator on cytotoxicity. While monotreatment with either cisplatin or 5′-Azacitidine (5-AZA) partially reduced tumor development, cotreatment of both drugs synergistically suppressed tumor development. Our results show that 5-AZA could indeed upregulate *ATM* by demethylating the *ATM* promoter, which consequently increased the vulnerability of EBV-infected cells to EBV lytic reactivation. Cotreatment of cisplatin and 5-AZA also accelerated apoptotic cell death in SNU719 cells compared to monotreatment. Both cisplatin and 5-AZA synergistically worked to remove in vitro EBV-infected cells and suppress in vivo tumor development. However, this synergistic effect in tumorigenesis was undermined by the loss of functional ATM. Interestingly, cotreatment of cisplatin and 5-AZA stimulated the development of the xenograft tumor in mice even though this stimulatory effect was insignificant. The loss of ATM clearly led to different cellular responses to cisplatin and 5-AZA, whose mechanism is left for further study.

**Figure 14:**
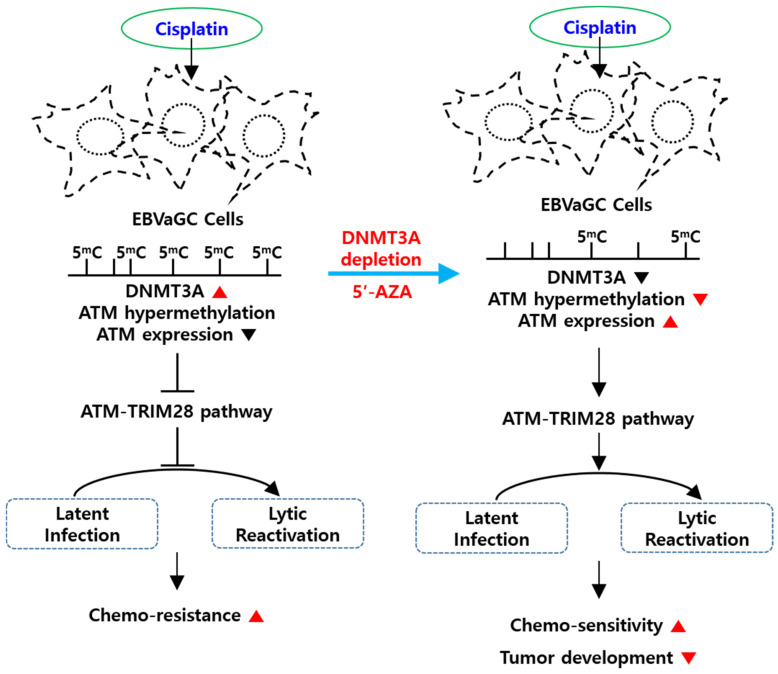
Schematic model for molecular mechanisms used by cisplatin for anticancer activity. Cisplatin monotherapy tends to upregulate the *DNMT3A* gene, downregulates the *ATM* gene, and fails to induce EBV lytic reactivation or produce chemo-resistance. However, cisplatin therapy in combination with 5-AZA tends to downregulate the *DNMT3A* gene, upregulate the *ATM* gene, induce EBV lytic reactivation, and eventually produce chemo-sensitivity.

The current study evaluated the clinical utility of cisplatin and 5-AZA cotreatment for EBVaGC. Our in vitro and in vivo experiments showed that DNA methylation inhibitors, such as 5-AZA, can work synergistically with cisplatin to maximize the effectiveness of cisplatin-based chemotherapy (Figure 14). These synergistic effects may help the development of a novel therapeutic approach for the treatment of EBVaGC. Several papers have been published that show that 5-AZA enhances the efficacy of cisplatin chemotherapy in lung and ovarian cancers [53,54], but not in gastric cancer. Thus, this study is the first to show the potential for cisplatin and 5-AZA cotreatment as an effective anti-gastric cancer approach in the near future.

## 4. Materials and Methods

### 4.1. Cell Lines and Reagents

Both gastric carcinoma cell lines SNU719 (EBVaGC) and MKN1 (EBVnGC) were purchased from Korean Cell Line Bank (Seoul, Korea) and cultured in RPMI 1640 (Hyclone, Pittsburgh, PA, USA) supplemented with 10% fetal bovine serum (FBS, Hyclone, Marlborough, MA, USA), antibiotics/antimycotics (Gibco, Waltham, MA, USA) and GlutaMAX (Gibco, Waltham, MA, USA) at 37 °C with 5% CO_2_ and 95% humidity. EBV BART^+^ bacmid was obtained from Dr. Teru Kanda [55]. MKN1 cells were transfected with EBV BART^+^ bacmid and selected with 30 μg/mL hygromycin B (Wako, Osaka, Japan) for at least 2 weeks. Resultant MKN1 cells were named MKN1–EBV, cultured in RPMI 1640, and supplemented with 10% FBS, antibiotics/antimycotics, and GlutaMAX. YECCL1 cells were cultured in EMEM (Lonza, Basel, Switzerlan) supplemented with 10% fetal bovine serum (FBS, Hyclone, Marlborough, MA, USA), antibiotics/antimycotics (Gibco, Waltham, MA, USA) and GlutaMAX (Gibco, Waltham, MA, USA) at 37 °C with 5% CO_2_ and 95% humidity.

### 4.2. Cytotoxicity Assay

The cytotoxic effects of cisplatin and 5′-Azacitidine (5-AZA) on SNU719 cells, MKN1 cells, MKN1–EBV cells, and YECCL1 cells were evaluated using Cell Counting Kit-8 (CCK-8; Dojindo, Kumamoto, Japan). Briefly, 100 μL of cell suspension (2 × 10^4^ cells/well) was seeded into a 96-well plate. The following day, cisplatin or 5-AZA was applied at various concentrations: 0.00–66.66 or 0.00–83.50 μM of cisplatin and 0.00–3275.9 μM of 5-AZA. After 48 h of treatment, 10 μL of CCK-8 solution was added to each sample. After incubating the samples for another 3 h, the absorbance of each cell suspension was measured at 450 nm using an enzyme-linked immunosorbent assay reader. All steps of the manufacturer’s recommended protocol were followed. Approximately 50% cytotoxicity (CD_50_) was determined as previously described [56]. Briefly, the middle absorbance between the highest and lowest absorbance was first calculated. Secondly, the concentration of the compound was evaluated by assigning a corresponding concentration to the middle absorbance. Thirdly, this compound concentration was identified as the CD_50_ concentration. In subsequent experiments, cells were treated with compounds at CD_50_ concentration for 48 h, after which old media containing mostly dead cells were removed, cells were further washed with phosphate-buffered saline at least twice to remove clearly dead cells, and finally 90% of live cells on average were harvested for analysis.

### 4.3. Luciferase Assay

SNU719 cells carrying the EBV BHLF1 promoter-luciferase construct were established and named SNU719-BHLF1 for subsequent luciferase assays [57]. SNU719-BHLF1 cells were treated with either cisplatin alone (0.00~21.10 μM) or in combination with 5-AZA (0.00~1.32 μM for cisplatin, 0.00~134.9 μM for 5-AZA) in various concentrations for 48 h. Luciferase activity was measured using a dual-luciferase reporter assay system (Promega, Madison, WI, USA) according to the manufacturer’s protocol.

### 4.4. Western Blot Assay

To assess the regulatory effects of cisplatin and 5-AZA on EBV protein synthesis, Western blotting was performed using SNU719 cells, MKN1 cells, MKN1–EBV cells, and YECCL1 cells treated with either cisplatin and 5-AZA alone or in combination. Treated cells were harvested using trypsin 48 h after treatment. Cells (10 × 10^6^) were lysed using 200 μL of radioimmunoprecipitation assay (RIPA) lysis buffer (Tris-HCl (50 mM, pH 8.0)), NaCl (150 mM), ethylenediaminetetraacetic acid (EDTA; 2 mM, pH 8.0), 1% NP-40, 0.5% sodium deoxycholate, and 0.1% sodium dodecyl sulfate (SDS)) and supplemented with proteinase inhibitor (PI, Sigma, St. Louis, MO, USA) and phenylmethylsulfonyl fluoride (PMSF, Sigma, St. Louis, MO, USA). Xenograft tissue samples were lysed using 500 μL of RIPA lysis buffer with PI and PMSF and then homogenized using pestles. Cell or tissue lysates were further fractionated using a Bioruptor Sonicator set to provide 30-s on/off pulses for 5 min (Cosmo Bio, Tokyo, Japan). Protein in the cell lysates was measured using the Bradford assay. Equivalent quantities of protein were separated in 10% SDS polyacrylamide electrophoresis gel and transferred to 0.45 μm polyvinylidene fluoride membranes (Millipore, Darmstadt, Germany). Membranes were probed with antibodies against EBV and cellular proteins. Thereafter, BZLF1 (Santa Cruz Biotechnology (SCB), Santa Cruz, CA, USA), EA-D (SCB), DNMT1 (Cell Signaling Technology (CST), Danvers, MA, USA), DNMT3A (CST), ATM (CST), TRIM28 (CST), and phospho-TRIM28 (Ser 824, CST) were detected using GAPDH (CST) and β-actin (SCB) as internal controls. goat-anti-mouse IgG-HRP (Genetex, Irvine, CA, USA) and goat-anti-rabbit IgG-HRP (Genetex, Irvine, CA, USA) were used as secondary antibodies. Antibody-bound proteins were visualized using an enhanced chemiluminescent detection reagent (GE Healthcare, Chicago, IL, USA). Membranes were stripped and reprobed with other antibodies.

### 4.5. Protein Stability Assay

To determine protein stability, SNU719 cells were treated with 100 μg/mL cycloheximide (CHX, Sigma, St. Louis, MO, USA) and 21.10 μM cisplatin for 0, 6, 12, 24, and 48 h. Treated cells were then harvested and prepared for Western blot assay to analyze DNMT3A stability. Intensity bands were analyzed using Image J software https://imagej.nih.gov/ij/docs/index.html (accessed on 12 May 2021)).

### 4.6. Lentiviral Transduction

pLKO.1 vector-based shRNA constructs for DNA methyltransferase 1 (TRCN 0000021891, TRCN 0000021892, and TRCN 0000021893 for DNMT1) and DNA methyltransferase 3A (TRCN 0000035756, TRCN 0000035757, and TRCN 0000035758 for DNMA3A) were purchased (Sigma, St. Louis, MO, USA). Control shRNA (shCTL) were generated in the pLKO.1 vector with the target sequence 5′-TTA TCG CGC ATA TCA CGC G-3′. Lentiviruses were produced using envelope and packaging vectors pMD2.G and pSPAX2 as described previously. SNU719 cells were infected with lentivirus stocks carrying pLKO.1-puro vectors by overlaying the lentivirus stock on SNU719 cells for 24 h. Thereafter, the lentivirus stocks were replaced with fresh RPMI medium and treated with 2.0 μg/mL puromycin 48 h after infection. The RPMI medium with 1.0 μg/mL puromycin was replaced every 2 to 3 days. Cells were selected using puromycin for at least 14 days. Resultant SNU719 cells were named as followed then subjected to further analyses: SNU719-pLKO.1 cells, SNU719-shDNMT1 cells, and SNU719-shDNMT3A cells.

### 4.7. Quantitative Reverse-Transcription Polymerase Chain Reaction Assay

Quantitative reverse-transcription polymerase chain reaction (qRT-PCR) assays were performed to quantify transcripts of interesting genes in SNU719 cells, MKN1 cells, MKN1–EBV cells, and YECCL1 cells. RNA was isolated from the cells using the RNeasy mini kit (Qiagen, Germantown, MD, USA). Purified RNA was converted into cDNA using SuperScript III Reverse Transcriptase (Invitrogen, Carlsbad, CA, USA). Diluted RT products were analyzed using real-time PCR (LightCycler 96, Roche, Basel, Switzerland). mRNA levels of actin or *GAPDH* in each sample were used as the internal control. qRT-PCR with non-reverse-transcribed RNA was conducted to serve as the negative control in each reaction. Gene primer sets specific to qRT-PCR were as follows: DNMT3A (forward: CCT GTG GGA GCC TCA ATG TTA, reverse: CTT GCA GTT TTG GCA CAT TCC) and ATM (forward: TTT ACC TAA CTG TGA GCT GTC TCC AT, reverse: ACT TCC GTA AGG CAT CGT AAC AC)

### 4.8. EBV Promoter Usage Assay

RNA was isolated from SNU719 cells using an RNeasy Mini Kit (Qiagen, Germantown, MA, USA), after which purified RNA was synthesized into cDNA using SuperScript III Reverse Transcriptase (Invitrogen, Carlsbad, CA, USA). Resultant cDNA was subjected to semi-quantitative PCR assay for the detection of EBV promoter activity. The effects of cisplatin on EBV promoter activity were then examined. Primer sequences for β-actin, EBV Qp, and EBV Fp were similar to those previously published. cDNA was amplified in a 25 μL reaction solution containing 2.5 μL of 10× reaction mix, 2.5 μL of TuneUp solution, 0.25 μL of Taq Plus polymerase, and 2.5 μL of 10 pmol forward/reverse primer. The following cycle conditions were used: 95 °C for 3 min; 30 cycles of 95 °C for 10 s, 55 °C for 30 s, and 72 °C for 30 s; and 72 °C for 10 min. Primers specific to the EBV promoters were as follows: Qp (F: 5′-GTG CGC TAC CGG ATG GC-3′, R: 5′-CAT GAT TCA CAC TTA AAG GAG ACG G-3′), and Fp (F: 5′-GGG TGA GGC CAC GCT TT-3′, R: 5′-CAG GTC TAC TGG CGG TCT ATG AT-3′). PCR reactions were subjected to a TaKaRa PCR Thermal Cycler (TaKaRa, Kyoto, Japan) and run on a 1.2% agarose/TBE gel.

### 4.9. Cell Viability Assay

Cell viability was determined using a MUSE count and viability kit (Merck Millipore, Darmstadt, Germany) according to the manufacturer’s protocol. Serval cells (1 × 10^6^) including SNU719 cells and MKN1–EBV cells were seeded into 6-well plates and treated with cisplatin or 5′ azacytidine for 24 or 48 h. A negative control sample was treated with the same volume of Dulbecco’s PBS (DPBS) for 24 or 48 h. Treated cells were trypsinized, harvested, and resuspended at 10^6^–10^7^ cells/mL of fresh serum. The cell suspension (20 μL) was mixed with 380 μL of the MUSE count and viability reagent and then incubated at 25 °C for 5 min. Cell viability was measured using MUSE cell analyzer (Millipore, Burlington, MA, USA).

### 4.10. Annexin V and Dead Cell Assay

To determine apoptotic cell death according to cisplatin treatment, a MUSE annexin V and dead cells assay kit (Merck Millipore, Darmstadt, Germany) was used. Serval cells (1 × 10^6^), such as SNU719-pLKO.1, SNU719-shDNMT1, and SNU719-shDNMT3A, were seeded into a 6-well plate and treated for 48 h with 1.32 μM and 21.1 μM of cisplatin for 48 h. A negative control sample was treated with the same volume of PBS for 48 h. Treated cells were trypsinized, harvested, and resuspended at 10^6^/mL in RPMI medium with FBS. The cell suspension (100 μL) was mixed with 100 μL of MUSE annexin V and dead cell reagent for 20 min at room temperature. Apoptosis was measured using the MUSE cell analyzer (Millipore, Burlington, MA, USA) according to the manufacturer’s protocol.

### 4.11. Methylated DNA Precipitation (MeDIP) Assay

A quantity of2’-deoxy-5′-azacytidine (DAC) was used to treat SNU719, SNU719-pLKO.1, and SNU719-shDNMT3A cells for 72 h. These cells were then subjected to genomic DNA (gDNA) isolation as follows: gDNA was isolated using the proteinase K and phenol/chloroform extraction protocol. Accordingly, 8 μg of gDNA was resuspended in up to 450 μL of 1× TE buffer (50 mM Tris buffer (pH 8.0) and 10 mM EDTA buffer) and denaturized by heating at 95 °C for 10 min followed by rapid chilling on ice. Thereafter, 1 μg of DNA was extracted to be used as the input control DNA, after which 45 μL of 10× immunoprecipitation buffer (100 mM sodium phosphate buffer (pH 7.0), 1.4 M NaCl, and 0.5% Triton X-100) was added. DNA samples were immunoprecipitated with 2 μg of anti-5mC (Active Motif, Carlsbad, USA) or 2 μg of mouse isotype anti-IgG control (Sigma-Aldrich) for 2 h at 4 °C with rotation. The antibody-bound DNA was captured with Dynabeads Protein G (ThermoFisher Scientific, Grand Island, NY, USA) for 2 h at 4 °C with rotation. The DNA captured beads were washed three times with 1× IP buffer for 10 min at room temperature with rotation and eluted in 250 μL of proteinase K digestion buffer (50 mM Tris buffer (pH 8.0), 10 mM EDTA buffer, 0.5% SDS, and 70 μg of proteinase K) at 50 °C for 3 h using a Thermomix C (Eppendorf, Hamburg, Germany) at 800 rpm. Eluted DNA samples were isolated with phenol/chloroform DNA purification and precipitated using 2 volumes of 100% EtOH, 400 mM NaCl, and 20 μg glycogen overnight at 4 °C. Precipitated DNA was measured using Nanodrop, and PCR was performed on 5 ng of DNA to determine ATM methylation, with EBV Cp and Qp as methylation controls. Conventional PCR assays were performed using a HelixAmp Taq Polymerase Kit (Nanohelix, Deajeon, Korea). The PCR cycle conditions and primer sets used are available upon request.

### 4.12. Bisulfite Conversion and Pyrosequencing Analysis

To analyze ATM 5′-UTR II methylation, SNU719 cells were treated with either DPBS or 1.32 μM cisplatin and SNU719-shDNMT3A cells were treated with 1.32 μM cisplatin for 48 h. gDNA was then isolated from these cells using the procedure described above. Resultant gDNA was further subjected to bisulfite modification using an EZ DNA methylation kit (Zymo Research, Orange, CA, USA) according to the manufacturer’s protocol. A total of 2 μg of gDNA was denatured by adding 5.5 μL of 2M NaOH at 37 °C for 10 min, after which 30 μL of 10 mM hydroquinone and 520 μL of 3M sodium bisulfite was added. The mixture was incubated at 50 °C for 17 h, desalted using the Wizard DNA Purification Resin Kit (Promega, Madison, WI, USA), and desulfonated by adding 5.5 μL of 3 M NaOH for 5 min. The modified DNA was precipitated with ethanol and resuspended in 35 μL of nuclease-free water. The PCR for the ATM 5′-UTR II was performed using the Solg™ h-Taq DNA Polymerase Kit (Solgent, Daegeon, Korea) with the following primer and cycle conditions: F: 5′-TGT TGT TTA GGT TGG AGT ATA GT-3′, R: 5′-biotin-ACC AAC ATA AAA CCC TAT CTC T-3′, sequencing primer: 5′-TTT TGA GTA GTT GGG ATT A-3′, and 95 °C for 15 min; 40 cycles of 95 °C for 55 s, 60 °C for 55 s, 72 °C for 60 s, and finally 72 °C for 10 min. PCR products were confirmed using agarose gel electrophoresis, while pyrosequencing analysis was performed using the PyroMark Q24 (Qiagen, Hilden, Germany). Methylated and unmethylated DNA were used as methylation and unmethylation controls, respectively.

### 4.13. Depletion of ATM Proteins via CRISPR/Cas9 Genomic Editing

To knock out *ATM* in SNU719 cells and MKN1–EBV cells, the CRISPR/Cas9 system was applied to these cells. A single-guide (sg) RNA sequence was designed using the web tool of the Centre for Organismal Studies, Heidelberg University (CCTop, https://crispr.cos.uni-heidelberg.de/ (accessed on 7 August 2021)). The following oligo set was used as sgRNA to target the *ATM*: forward oligo 5′-AAAc TGA GTC TAG TAC TTA ATG ATC-3′, reverse oligo 5′-CACCg ATC ATT AAG TAC TAG ACT CA-3′. The sgRNA targeting human ATM (NG_009830.1) was designed and cloned into the LentiCRISPRv2 vector (Addgene number 52961). The LentiCRISPRv2 vector or LentiCRISPRv2-ATM sgRNA vector was co-transfected with the lentivirus packaging plasmids (psPAX2, pMD2.G) into HEK 293T cells for 72 h. The viral supernatants were then harvested, filtered, and used to infect SNU 719 cells and MKN1–EBV cells for 24 h. Thereafter, the viral supernatants were replaced with fresh RPMI medium and treated with 0.5 μg/mL puromycin every 2 to 3 days, after which knockout efficiency was confirmed through protein expression. Finally, resultant SNU719 cells were named SNU719-C/Cas9 and SNU719-ATM(-) cells, and the resultant MKN1–EBV cells named MKN1–EBV-C/Cas9 and MKN1–EBV-ATM(-) cells.

### 4.14. Anti-Tumor Assay in a Xenograft Mouse Model

Nude mice (female, 5-week-old; Raonbio Co., Ltd., Seoul, Korea) were used to establish xenograft animal models to assess anti-tumor effects. Mice were individually housed in a pathogen-free controlled environment (23–27 °C under a 12-h light/dark cycle) and provided food and water ad libitum. At first, we evaluated the synergistic anti-tumor effect of a combination therapy of cisplatin and 5-AZA. To this end, 2.5 × 10^6^ MKN1–EBV cells were subcutaneously implanted into the dorsum next to the right hind leg of the mice (*n* = 35). After 14 days, xenograft mice were randomly divided into four groups that received control (PBS, *n* = 8), cisplatin (0.5 mg/kg, *n* = 9), 5-AZA (2 mg/kg, *n* = 9), and combination (0.5 mg/kg cisplatin and 2 mg/kg 5-AZA, *n* = 9) for 43 days. Animals in each group received the same volume of PBS, cisplatin, 5-AZA, and combination therapy via weekly intraperitoneal injection. Tumors were identified and measured every other day using a standard caliper, while tumor size was calculated using the following formula: [tumor length (mm) × tumor width (mm)^2^]/2. After the tumor size had reached 1000 mm^3^, the animals were euthanized, and tumors were harvested. The animal experiments were conducted in accordance with the National Research Council’s Guide (IACUC, Seoul, Korea) for the Care and Use of Laboratory Animals. The experimental protocol was approved by the Animal Experiments Committee of Duksung Women’s University (permit number: 2019-003-004).

Subsequently, we investigated the necessity of functional ATM for producing a synergistic anti-tumor effect in combination therapy with cisplatin and 5-AZA. To this aim, 2.5 × 10^6^ MKN1–EBV-ATM(-) cells were subcutaneously implanted into the dorsum next to the right hind leg of the mice (*n* = 32). After 14 days, xenograft mice were randomly divided into four groups that received control (PBS, *n* = 8), cisplatin (0.5 mg/kg, *n* = 8), 5-AZA (2 mg/kg, *n* = 8), and combination (0.5 mg/kg cisplatin and 2 mg/kg 5-AZA, *n* = 8) for 33 days. Similarly to the previous anti-tumor assay using MKN1–EBV cells, animals in each group received the same volume of PBS, cisplatin, 5-AZA, and combination via weekly intraperitoneal injection. Tumors were identified and measured every other day by same methods. After the tumor size had reached approximately 1000 mm^3^, the animals were euthanized, and tumors were harvested. The animal experiments were conducted in accordance with the National Research Council’s Guide (IACUC, Seoul, Korea) for the Care and Use of Laboratory Animals. The experimental protocol was approved by the Animal Experiments Committee of Duksung Women’s University (permit number: 2020-007-004).

### 4.15. IHC Analysis

The tumor tissues were fixed in 4% paraformaldehyde and embedded in paraffin. Subsequently, paraffin sections were performed according to the manufacturer’s protocol (Abcam, Cambridge, UK). Deparaffinized and rehydrated 7-μm sections were immunostained with the antibodies against EBNA1 (Santa Cruz). After development with a peroxidase reagent, diaminobenzidine (Vector, Linaris, Germany) and counterstaining with hematoxylin, stained tumor tissues were visualized by light microscopy.

### 4.16. Kaplan–Meier Analysis

The 5-year overall survival (OS, *n* = 881) rates were analyzed for gastric cancer cases from the Kaplan–Meier plotter [43]. A total of 320 patients were analyzed from the following datasets: GSE14210, GSE15459, GSE22377, GSE29272, GSE51105, and GSE62254. Input genes were DNMT3A (Affy id: 222640_at) and ATM (Affy id: 212672_at). These analyses were restricted to Lauren classification intestinal (*n* = 336) types of gastric cancers. Other subtypes included all patients (gender, perforation, treatment, and HER2 status).

### 4.17. Statistical Analysis

Statistical analyses were conducted using a two-tailed Student’s *t*-test (Microsoft, Redmond, WA, USA) and a one-way analysis of variance (ANOVA) and re-verified thorough Dunnett’s multiple comparison test using GraphPad Prism (San Diego, CA, USA).

## Data Availability

The data are publicly available.

## Supplementary Materials

The following are available online at https://www.mdpi.com/article/10.3390/cancers13174252/s1, Figure S1: Prediction of DNA hypermethylation on ATM 5’-UTR. Bioinformatical analysis of CpG di-nucleotide in ATM 5’ UTR (NC_000011.10) using MEXPRESS tool. Figure S2: The measurement of DAB (3, 3′-diaminobenzidine) level in the IHC sample sections from MKN1-EBV-ATM(-) cell xenograft mice. DAB-positive spot (DPS)s were counted at each of the eight locations origi-nating from two sections by using the ImageJ software. Next, we statistically processed the DPS numbers to determine the differences in the sample groups by using ANOVA and Dunnett’s Multiple Comparison Test.
