# Supplementary material for: Cisplatin Resistance in Epstein–Barr-Virus-Associated Gastric Carcinoma Acquired through ATM Methylation
Source: Cancers (Basel). 2021 Aug 24;13(17):4252. doi: 10.3390/cancers13174252 (PMC8428228; doi:10.3390/cancers13174252)
Supplement: Supplementary file 1 [file cancers-13-04252-s001.zip › cancers-1339391-supplementary.pdf]

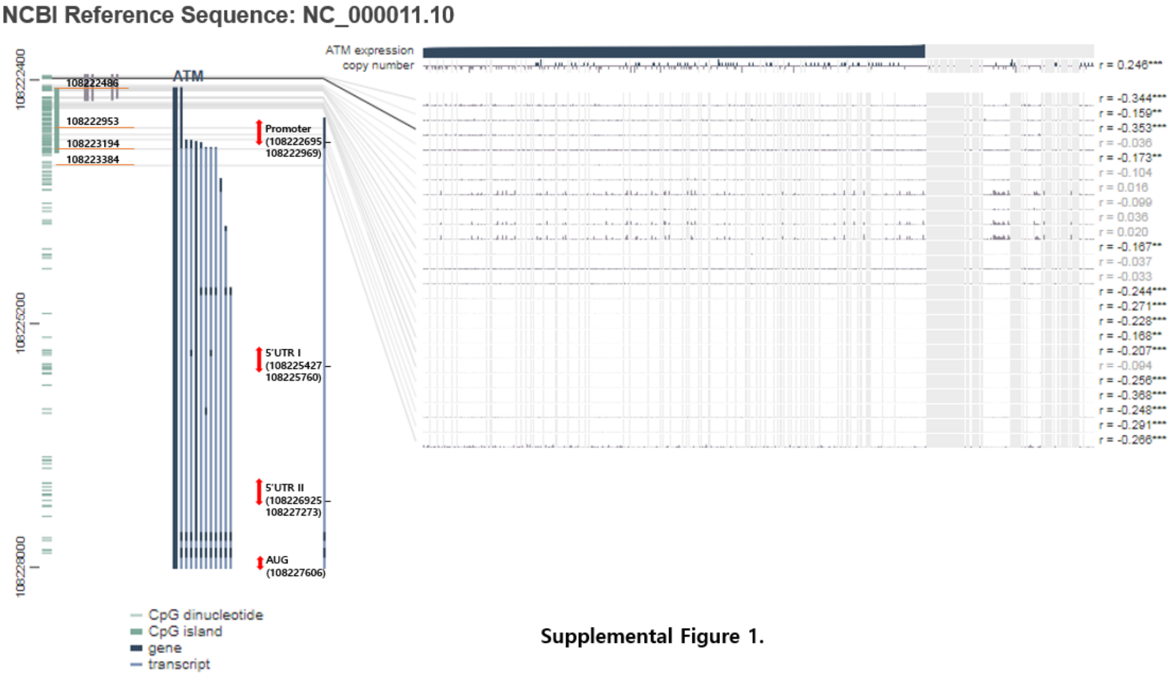

Supplemental Figure S1. Prediction of DNA hypermethylation on ATM 5′-UTR. Bioinformatical analysis of CpG dinucleotide in ATM 5′ UTR (NC_000011.10) using MEXPRESS tool.

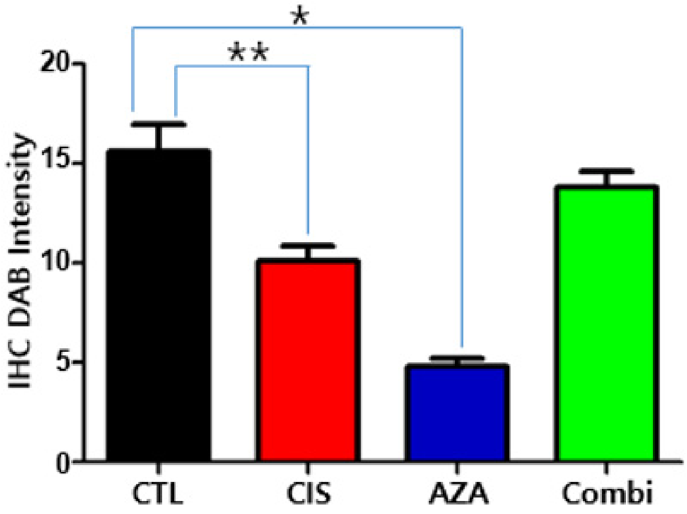

Supplemental Figure S2. The measurement of DAB (3, 3′-diaminobenzidine) level in the IHC sample sections from MKN1-EBV-ATM(-) cell xenograft mice. DAB-positive spot (DPS)s were counted at each of the eight locations originating from two sections by using the ImageJ software. Next, we statistically processed the DPS numbers to determine the differences in the sample groups by using ANOVA and Dunnett's Multiple Comparison Test. (*, ** $p < 0.05$).
